# Radiological Characteristics of Carbonated Portland Cement Mortars Made with GGBFS

**DOI:** 10.3390/ma15093395

**Published:** 2022-05-09

**Authors:** Miguel Ángel Sanjuán, José Antonio Suárez-Navarro, Cristina Argiz, Marta Barragán, Guillermo Hernáiz, Miriam Cortecero, Pedro Lorca

**Affiliations:** 1 Spanish Institute for Cement and Its Applications (IECA), C/José Abascal, 53, 28003 Madrid, Spain; 2 Department of Environment, Environmental Radioactivity and Radiological Surveillance (CIEMAT), Avenida Complutense 40, 28040 Madrid, Spain; ja.suarez@ciemat.es (J.A.S.-N.); marta.barragan@ciemat.es (M.B.); guillermo.Hernaiz@ciemat.es (G.H.); 3 Department of Chemistry of Building Materials, Civil Engineering School, Technical University of Madrid (UPM), Ciudad Universitaria, C/Profesor Aranguren, s/n, 28040 Madrid, Spain; cg.argiz@upm.es; 4 IES Virgen de la Paloma, C/de Francos Rodríguez, 106, 28039 Madrid, Spain; miriamcortecero63@gmail.com (M.C.); pedrolorcasanchez@gmail.com (P.L.)

**Keywords:** mortar, ground granulated blast-furnace slag, natural radioactivity, microstructure, gamma spectrometry, radon emanation rate

## Abstract

The objective of this study is to assess whether the carbonation process can modify the physicochemical characteristics of the natural radionuclides of the three natural radioactive series, together with ^40^K. Three mortar specimens with different percentages of ground granulated blast-furnace slag (GGBFS), cured under water for 1, 3, 7, 14, or 28 days, were subjected to a natural carbonation process. Activity concentrations for the solid and ground mortars were determined by gamma spectrometry and by radiochemical separation of isotopic uranium. The novelty of this paper relies principally on the study we have carried out, for the first time, of the radiological characteristics of carbonated Portland cement mortars. It was found that the chemical properties of the 3 mortar specimens were not affected by the carbonation process, with particular attention placed on uranium (^238^U, ^235^U, and ^234^U), the activity concentrations of which were equivalent to the ^226^Ra results and ranged from 5.5 ± 1.6 Bq kg^−1^ to 21.4 ± 1.2 Bq kg^−1^ for the ^238^U. The average activity concentrations for the 3 types of mortars were lower than 20.1 Bq kg^−1^, 14.5 Bq kg^−1^, and 120.2 Bq kg^−1^ for the ^226^Ra, ^232^Th (^212^Pb), and ^40^K, respectively. Annual effective dose rates were equivalent to the natural background of 0.024 mSv. In addition, it was observed that the variation rate for the ^222^Rn emanation was due primarily to the Portland cement hydration and not due to the pore size redistribution as a consequence of the carbonation process. This research will provide new insights into the potential radiological risk from carbonated cement-based materials. Moreover, the assessment that is presented in this study will convey valuable information for future research that will explore the activity concentration of building materials containing NORM materials.

## 1. Introduction

In a previous work, the authors studied the carbonation process in mortars made with ground granulated blast-furnace slag (GGBFS) cements [1]. The results showed that the degree of carbonation increased proportionally with the ground granulated blast-furnace slag content and inversely proportional to the curing time. The samples selected to perform this study were taken from a former research program on the durability of cement-based materials published in [1]. However, the aims and objectives of the former study and the present one are completely different, i.e., focussing on durability and radioactivity.

Therefore, this study is intended as a continuation of the previous paper in pursuing the physicochemical behaviour of radionuclides belonging to the three native radioactive series, together with ^40^K, which are present in the same mortars, as well as to assess the level of compliance with the European Council Directive 2013/59/Euratom [2].

Alternative raw materials that come from industrial waste are the key resources of a competitive economy based on the circular economy; therefore, as is well known, there are some modern trends in the use of waste from various industries in the building materials sector. Amran et al. reviewed coal-fly-ash-based concretes [3]. They concluded that the use of coal fly ash as a supplementary cementitious material can mitigate the carbon dioxide emissions in the cement manufacture and the health and disposal issues. Finally, they suggest that the use of coal fly ash, as well as the utilization of ground granulated blast-furnace slag (GGBFS), should be highlighted in the design of eco-friendly buildings and cities. In addition, Volodchenko et al. [4] remark that the decrease in fuel consumption minimizes the emission of carbon dioxide into the atmosphere and reduces the pollution of water, soil, and air. Therefore, the development of new strategies and the implementation of energy-saving technologies for the manufacture of building materials belong to the modern trends of “green” technologies.

The carbonation process consists of the formation of CaCO_3_ via the reaction of CO_2_ with the calcium phases present in Portland-cement-based materials and H_2_O [5]. Carbonation starts with the dissolution of gaseous CO_2_ in the pore solution present in the capillary pores of mortars and concretes, which reacts mainly with Ca(OH)_2_ according to the following reaction [6]:

Ca(OH)_2_ + CO_2_ → CaCO_3_ + H_2_O (1)

Likewise, CO_2_ can react with calcium silicate hydrate (C-S-H) by the following reaction [7]:

a = 1, (3CaO·2SiO_2_·3H_2_O) + 3CO_2_ → 3CaCO_3_ + 2SiO_2_·nH_2_O + (3−n)H_2_O (2)

On the other hand, the clinker can also undergo the carbonation reactions of the 2CaO·SiO_2_ (C_2_S, dicalcium silicate) and 3CaO·SiO_2_ (C_3_S, tricalcium silicate) [8]:

2CaO·SiO_2_ + 2CO_2_ + nH_2_O → SiO_2_·nH_2_O + 3CaCO_3_ (3)

3CaO·SiO_2_ + 3CO_2_ + nH_2_O → SiO_2_·nH_2_O + 3CaCO_3_ (4)

Council Directive 2013/59/Euratom [2], which lays down basic safety standards for protection against the dangers arising from exposure to ionising radiation, states that the reference level applying to indoor external exposure to gamma radiation emitted by building materials shall be 1 mSv per year (Article 75: “Gamma radiation from building materials”). The effective dose is determined by the absorbed dose from a standard room model of 2.8 m × 4 m × 5 m, assuming a wall thickness of 20 cm with a density of 2.35 g cm^−3^. This effective dose rate was determined by suppressing the absorbed dose due to the background (50 nGy h^−1^ in the case of Europe) and taking into account the activity concentrations of ^226^Ra, ^232^Th, and ^40^K. In the case of ^226^Ra, that would take into account the natural radionuclides belonging to the natural radioactive series of uranium and, particularly, ^226^Ra and its radioactive progeny. In the uranium radioactive series, carbonation could significantly affect the parent of the series (^238^U), ^234^U, and ^222^Rn, which is gaseous. Uranium in uranyl form (UO_2_^2+^) produces highly stable complexes with carbonate [9]. These complexes are soluble and enhance the release of uranium from different types of minerals [10]. In this sense, CaCO_3_ generated by Portland cement carbonation has been shown, in several laboratory tests and mathematical models, to increase uranium mobility due to the formation of the calcium-uranyl-carbonate complexes Ca_2_UO_2_(CO_3_)_3_^0^ and CaUO_2_(CO_3_)_3_^2−^ [11].

Carbonation also produces a narrower pore-size distribution. It depends on the cement type [12]. These microstructural changes could influence the ^222^Rn emanation and exhalation in these carbonated materials [13]. On the other hand, radon emanation would be influenced by the distribution of ^226^Ra atoms and the internal microstructure of the material [14], which is altered by the carbonation process. Therefore, this paper studies the effect of the carbonation process on the activity concentration of natural radionuclides, paying special attention to uranium and radon, in different Portland cement mortars made with Portland cements with two percentages of ground granulated blast-furnace slag (GGBFS).

Our hypothesis is that the carbonation process does not influence, either chemically or physically, the radiological properties of mortars made with Portland cements containing different percentages of ground granulated blast-furnace slag. The hypothesis was statistically tested by comparing the activity concentrations of carbonated and non-carbonated mortars made with 3 types of Portland cements: CEM I 52.5 R-SR 3, CEM II/A-S 42.5N, and CEM III/A 42.5N. The ground granulated blast-furnace slag content was 0%, 14.4%, and 28.1%, respectively.

The primary objectives of this study were: (i) to compare the activity concentrations of the natural radionuclides present in the hardened mortar specimens (40 mm side) and in the ground mortars; (ii) to study the influence of the carbonation process on the activity concentration of the radionuclides present in the ground mortars; and (iii) to determine whether the carbonation process influences the annual effective dose rates by external irradiation and the ^222^Rn emanation power of mortars. 

## 2. Materials and Methods

### 2.1. Raw Materials for Mortar Manufacturing 

A total of 3 Portland cements, according to EN 197-1:2011 [15], were tested in this study. CEM I 52.5 R-SR (C1), without additions, and the rest of the cements contain several of the contents of ground granulated blast-furnace slag (CEM II/A-S 42.5 N (C2): 14.4% and CEM III/A 42.5 N (C3) with 28.1%. Chemical analyses (Table 1) were carried out according to EN 196-2:2014 [16].

The physicochemical properties of these cements were presented in a previous study [1]. Moreover, a European standard EN 196.1-2018-compliant standardised sand was used as an aggregate [17,18].

### 2.2. Design and Manufacturing of Test Samples

The mortar samples have cement/sand and water/cement ratios of 1/3 and 0.50, respectively. They were cured at 99% relative humidity for 1 day. Later, they were demolded and cured for 0, 1, 3, 7, 14, or 28 days under water. Finally, the mortar specimens were submitted to natural carbonation [16]. Natural carbonation testing was performed following the Technical Specification CEN/TS 12390-10 [19]. Mortar specimens were exposed to an outdoor environment sheltered from rain conditions (about 0.035 ± 0.005%, 20 ± 2 °C and 65 ± 5% RH). Carbonation depth measurements were taken at 24 months of natural exposure after the completion of the under-water curing period. The mortar prisms were sawn up, and the cleaned surface was sprayed with a pH-indicator solution prepared by dissolving 1 g of phenolphthalein in a 100 mL solution (70 mL ethanol plus 30 mL distilled water) [1]. Afterwards, gamma spectrometry measurements were carried out on the fully carbonated mortars.

Two types of samples were used in this study: (i) quadrangular prisms resulting from the strength tests (4 cm on each side and between 2 and 3.8 cm in height) and (ii) solid samples in powder form, created by the grinding of the prisms. A Retsch ball-mill, model “Pulverisette 5” (Haan, Germany), was used for the grinding operation. Later on, the powder was dried for 24 h at 105 °C in a Selecta oven (Abrera, Spain) and sieved at 250 µm. The sample was placed in a cylindrical, polypropylene box, 30 mm high and 76 mm in diameter.

### 2.3. Gamma Spectrometry Measurements

The laboratory in which the gamma spectrometry measurements were performed is accredited according to UNE-EN ISO/IEC 17025:2017 standard [20].

The high-purity germanium detectors used were 3, 2-coaxial detectors composed of *n*-type of material and a third broad-energy detector composed of *p*-type material; all the three detectors were manufactured by Canberra Industries (Canberra Industries, Meriden, Connecticut). The detectors were located in 15-centimeter-thick passive shielding made of Fe and Pb. All detectors had a resolution lower than 2 keV for the 1.33 MeV photopeak of ^60^Co.

The detectors were connected to associated electronics consisting of a high voltage source, an amplifier, an analogue-to-digital converter, and a communications module. The three detectors were adapted in the factory such that they would be able to use the LabSOCS software [21,22]. The photopeaks used to determine the activity concentrations of the radionuclides belonging to the three natural radioactive series (uranium, actinium, and thorium) and ^40^K were [23]: ^234^Th (63.30 (2) keV), ^226^Ra (186.211 (13) keV), ^214^Pb (351.932 (2) keV), ^214^Bi (609.312 (7) keV; 1120.287 (10) keV; 1764.494 (14) keV), ^210^Pb (46.539 (1) keV), ^212^Pb (238.632 (2) keV), ^208^Tl (583.187 (2) keV, ^228^Ac (911.196 (6) keV), ^235^U (163.356 (3) keV; 205.16 (4) keV; 143.767 (3) keV), and ^40^K (1460.822 keV). Interferences due to ^235^U in the 186 keV photopeak, in the determination of the ^226^Ra activity concentration, and ^228^Ac in the 1460 keV photopeak, in the determination of the ^40^K activity concentration were corrected using the algorithm described in [24].

The samples measured by gamma spectrometry are given in Section 2.2. The samples were measured for 80,000 s. The efficiencies of the quadrangular prisms and cylindrical boxes were calculated with LabSOCS software (Canberra Industries, Meriden, Connecticut) following the procedure described in [25]. The recording of the spectra and their subsequent analysis was performed using Genie 2000 software [26].

### 2.4. Determination of Isotopic Uranium Activity Concentration

The activity concentrations of ^238^U, ^235^U, and ^234^U were determined by a radiochemical method based on that proposed in [27]. The method consisted of a liquid–liquid extraction with ethyl acetate in a strongly saline medium composed of aluminium nitrate and tartaric acid. Subsequently, the uranium was electrodeposited following the Hallstadius method [28]. Finally, the samples were measured in a Canberra Industries Alpha Analyst device with 12 vacuum chambers containing the implanted silicon semiconductor detector.

The activity concentrations of ^238^U, ^235^U and ^234^U were determined by the following expression:

(5) $$A_{A_{U}}=\frac{c_{A_{U}}-\left(cb_{A_{U}}\cdot \frac{t_{m}}{t_{b}}\right)}{c_{232_{U}}-\left(cb_{232_{U}}\cdot \frac{t_{m}}{t_{b}}\right)}\cdot A_{232_{U}}\cdot V_{232_{U}}\cdot \frac{1}{M}$$

where, $A_{A_{U}}$ is the activity concentration of the uranium isotope of atomic mass *A*, in the units in which the sample quantity *M* is expressed; $c_{A_{U}}$ are the total counts in the uranium isotope peak of atomic mass *A*, in counts; $cb_{A_{U}}$ are the total counts due to the target for the uranium isotope of atomic mass *A*, in counts; $t_{m}$ is the sample counting time, in seconds; $t_{b}$ is the counting time of the blank, in seconds; $c_{232_{U}}$ are the total counts at the peak of ^232^*U*, in counts; $cb_{232_{U}}$ are the total counts of the blank for the ^232^*U* peak, in counts; $A_{232_{U}}$ reference activity of ^232^*U* corrected to the date of measurement, in becquerels per millilitre; $V_{232_{U}}$ is the volume of tracer added to the sample, in millilitres; M is the quantity of sample, expressed in units of mass or volume.

The uncertainty associated with the uranium activity concentration $\left(u\left(A_{A_{U}}\right)\right)$ was estimated by Equation (6):

(6) $$u\left(A_{A_{U}}\right)=A_{A_{U}}\cdot \sqrt{\frac{\left(c_{A_{U}}+{\left(\frac{t_{m}}{t_{b}}\right)}^{2}\cdot cb_{A_{U}}\right)}{{\left(c_{A_{U}}-cb_{A_{U}}\cdot \left(\frac{t_{m}}{t_{b}}\right)\right)}^{2}}+{\left(\frac{u\left(M\right)}{M}\right)}^{2}+{\left(\frac{u\left(V_{232_{U}}\right)}{V_{232_{U}}}\right)}^{2}+{\left(\frac{u\left(A_{232_{U}}\right)}{A_{232_{U}}}\right)}^{2}+\frac{\left(c_{232_{U}}+{\left(\frac{t_{m}}{t_{b}}\right)}^{2}\cdot cb_{232_{U}}\right)}{{\left(c_{232_{U}}-\left(\frac{t_{m}}{t_{b}}\right)\cdot cb_{232_{U}}\right)}^{2}}}$$

Finally, the detection limit was calculated by first determining the critical limit using the following Equation (7):

(7) $$A_{A_{U}}^{*}=k\cdot \frac{A_{232_{U}}\cdot V_{232_{U}}}{c_{232_{U}}-\left(cb_{232_{U}}\cdot \frac{t_{m}}{t_{b}}\right)}\cdot \frac{1}{M}\cdot t_{m}\cdot \sqrt{cb_{A_{U}}\cdot \left(\frac{1}{t_{m}\cdot t_{b}}\right)\cdot \left(1+\left(\frac{t_{m}}{t_{b}}\right)\right)}$$

where *k* is equal to 1.65, and the detection limit is given by Expression (8):

(8) $$A_{A_{U}}^{\#}=\frac{2\cdot A_{A_{U}}^{*}+\frac{A_{232_{U}}\cdot V_{232_{U}}}{c_{232_{U}}-(cb_{232_{U}}\cdot \frac{t_{m}}{t_{b}})}\cdot \frac{1}{M}\cdot k^{2}}{1-k^{2}\cdot \left({\left(\frac{u\left(M\right)}{M}\right)}^{2}+{\left(\frac{u\left(V_{232_{U}}\right)}{V_{232_{U}}}\right)}^{2}+{\left(\frac{u\left(A_{232_{U}}\right)}{A_{232_{U}}}\right)}^{2}+\frac{\left(c_{232_{U}}+{\left(\frac{t_{m}}{t_{b}}\right)}^{2}\cdot cb_{232_{U}}\right)}{{\left(c_{232_{U}}-\left(\frac{t_{m}}{t_{b}}\right)\cdot cb_{232_{U}}\right)}^{2}}\right)}$$

### 2.5. Determination of the Effective Dose and Emanation Factor of Radon 

The annual effective dose rate excess due to external gamma radiation for the model of a standard room of 2.8 m × 4 m × 5 m, with a wall thickness of 20 cm, with a density of 2.35 g cm^−3^, was determined by first calculating the absorbed dose rate (${\dot{D}}_{\gamma }$*,* nGy h^−1^) with Equation (9):

(9) $${\dot{D}}_{\gamma }=0.08\cdot C_{40_{K}}+0.92\cdot C_{226_{R}a}+1.1\cdot C_{232_{Th}}$$

where $C_{40_{K}}$, $C_{226_{R}a}$, and $C_{232_{Th}}$ are the activity concentrations (Bq kg^−1^) for the ^40^K, ^226^Ra, and ^232^Th (from the ^212^Pb), respectively. The uncertainty associated with Equation (9) is given by Equation (10):

(10) $$u\left({\dot{D}}_{\gamma }\right)=\sqrt{{\left(0.10\right)}^{2}\cdot u^{2}\left(C_{40_{K}}\right)+{\left(1.21\right)}^{2}\cdot u^{2}\left(C_{226_{R}a}\right)+{\left(1.29\right)}^{2}\cdot u^{2}\left(C_{232_{Th}}\right)}$$

The effective dose rate (E) is given by the following Equation (11) [29]:

(11) $$E={\dot{D}}_{\gamma }\cdot V\cdot T_{e}\cdot O\cdot 10^{-6}$$

where V is the factor to convert the absorbed dose into effective dose (0.7 Sv per Gy), $T_{e}$ is the number of hours per year (8760 h), O is the occupancy factor (0.8), and $10^{-6}$ is the factor to transform nano to milli. The uncertainty associated with Equation (11) is given by Equation (12):

(12) $$u\left(E_{P}\right)={\dot{D}}_{\gamma }\cdot V\cdot T_{e}\cdot O\cdot 10^{-6}\cdot u\left({\dot{D}}_{\gamma }\right)$$

The emanation power or emanation rate of a material is defined as the ratio between the radon activity emanated from the solid phase and the total radon activity concentration at equilibrium [30,31]. These concentrations can be determined by performing the measurement in the interior of an isolation chamber. In addition, radon concentration is measured with equipment such as AlphaGuard [32]; by alpha spectrometric detectors, such as a RAD7, which uses a solid-state alpha detector (usually silicon) [33]; or by a scintillation cell 300A [34].

These concentrations can also be measured by gamma spectrometry to determine the radon de-emanation or regrowth [30,35,36], or by using the theoretical radon growth curves [37,38]. In this work, the Pakov method [39], based on gamma spectrometric measurements, was selected. However, unlike the previously mentioned methods, it only requires one measurement whereby the activity concentration of ^226^Ra and the activity concentration of the ^222^Rn progeny (^214^Pb and ^214^Bi) is determined. In this method, the ^222^Rn emanation rate is determined by Equation (13):

(13) $$\varepsilon =\frac{C_{226_{R}a}-C_{214_{P}b}}{C_{226_{R}a}}$$

where $C_{214_{P}b}$ is the activity concentrations (Bq kg^−1^) for the ^214^Pb. The uncertainty associated with Equation (13) is given by Equation (14):

(14) $$u\left(\varepsilon \right)=\varepsilon \cdot \sqrt{2\cdot {\left(\frac{u\left(C_{226_{R}a}\right)}{C_{226_{R}a}}\right)}_{\;}^{2}+{\left(\frac{u\left(C_{214_{P}b}\right)}{C_{214_{P}b}}\right)}_{\;}^{2}}$$

### 2.6. Statistical Analysis 

The statistical analysis of the results of this study was performed with Statgraphics Centurion XVII version 17.0.16 (Statpoint Technologies Inc., The Plains, VA, USA) and Microsoft Excel (Redmond, WA, USA). The analyses performed were:

(a) Comparison of the mean and the dispersion values between the activity concentrations of the different natural radionuclides measured in the quadrangular, solid cement mortars and the ones measured in the powdered samples for the various curing times. Average values were compared by Student’s *t*-test for paired results and the dispersion by Fisher’s F-test. Both tests were performed using the two-tailed test as it was intended to assess whether or not the results show significant differences in both positive and negative skewness. Significant differences in accuracy (mean) and precision (dispersion) would occur for probability values below α = 0.05 [40,41].
(b) Comparison between the activity concentrations of non-carbonated and carbonated samples. The comparison was made by analysing the distribution of activity concentrations of ground non-carbonated and carbonated samples. The parameters used were kurtosis, relative skewness, coefficient of variation, and the Shapiro–Wilk normality test. The kurtosis and relative skewness values of a normal distribution must be between −2 and +2. Likewise, a normal distribution requires that the *p*-value obtained for the Shapiro–Wilk test is greater than the decision level (α = 0.05). The Shapiro–Wilk test is applicable to result sets between 3 and 50 [42]. Outliers were determined from the box-and-whisker plots.
(c) Comparison of the activity concentration distributions of each of the different natural radionuclides for ground carbonated materials at different curing ages.

## 3. Results

### 3.1. Radiological Characterisation of Quadrangular Mortar Prisms and Ground Mortars

Table 1 shows the activity concentrations obtained by gamma spectrometry for the radionuclides belonging to the three native radioactive series together with the ^40^K for the quadrangular prisms of hardened mortars. For each of the cements, the activity concentrations of the mortars, non-carbonated and carbonated, are presented. Table 2 shows the activity concentrations for the mortars shown in Table 1 as measured in powder form after grinding. On the other hand, the activity concentrations of ^238^U, ^235^U, and ^234^U determined by radiochemical separation are presented in Table 3.

### 3.2. Comparison between the Radioactive Content of Solid and Ground Mortars

**Figure 1:**
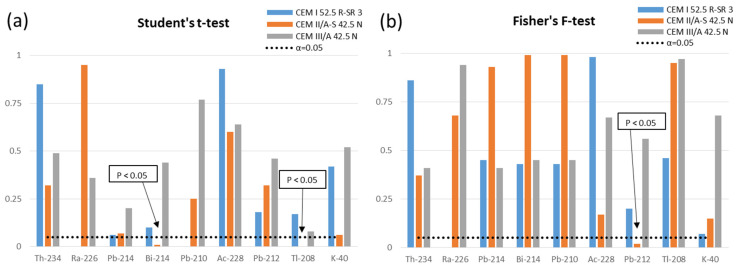
(**a**,**b**) Results of the evaluation of the mean (Student’s *t*-test) and variance (Fisher’s F-test) between the solid and ground mortars.

Figure 1 shows the values obtained from the Student’s *t*-test and Fisher’s F-test used to compare the mean and variance values of the activity concentrations of the solid and powdered mortars after grinding. The values for which the *p*-parameter was less than the chosen significance value (α = 0.05) have been indicated in the graph. These values would show statistical differences between means or variances. The radionuclides whose means were significantly different were: ^208^Tl and ^214^Bi for the mortar made with CEM II/A-S 42.5 N cement. On the other hand, the variances were not comparable for ^212^Pb for the mortar made with CEM II/A-S 42.5 N cement.

### 3.3. Influence of Carbonation on the Activity Concentration of the Studied Radionuclides

**Figure 2:**
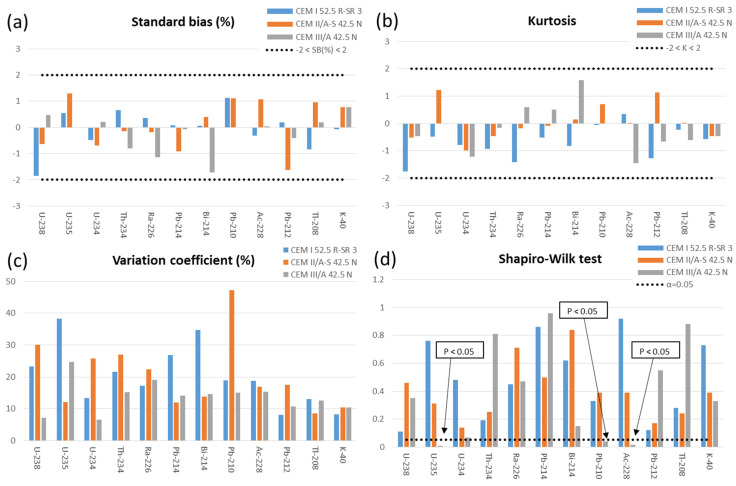
Statistical parameters obtained in the comparison of activity concentrations of carbonated and non-carbonated ground mortars: (**a**) SB standard bias (%), (**b**) K kurtosis, (**c**) variation coefficient (%), and (**d**) Shapiro–Wilk test statistic for normality.

Figure 2 shows the statistical parameters obtained in the comparison of the activity concentrations of the studied radionuclides for the carbonated and non-carbonated powder samples. The mortar carbonation process easily induces a decrease in the capillary pore system since products from the reaction between hydration products and carbon dioxide in the mortar fill in some of the pores, which further increases the mortar density and decreases the carbon dioxide diffusion coefficient in the concrete. Therefore, a pore-size redistribution is expected as consequence of the carbonation process [43].

**Figure 3:**
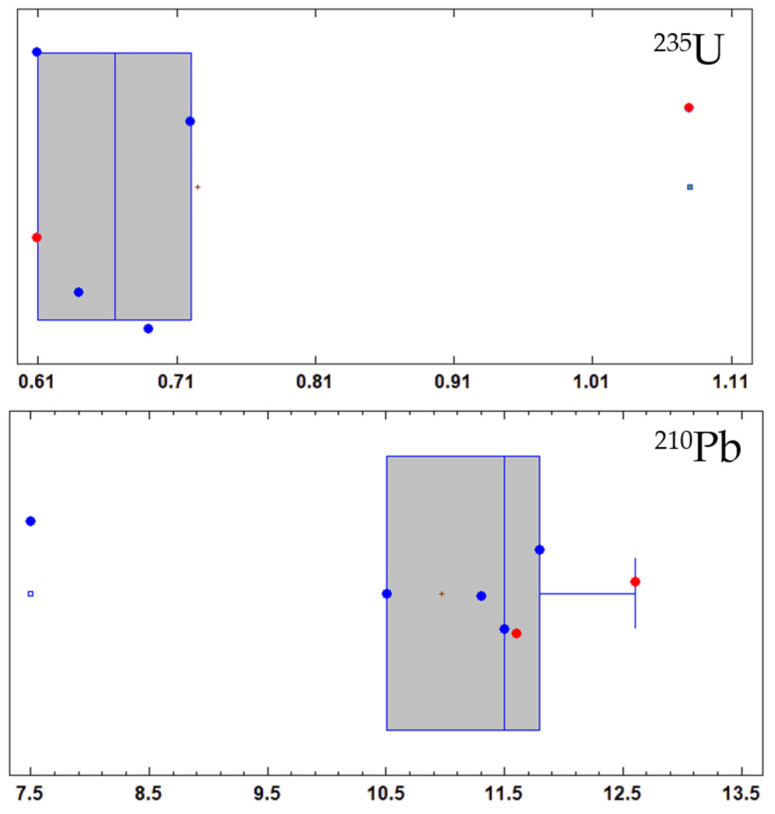
Box-and-whisker plots for ^235^U and ^210^Pb activity concentrations data recorded in ground mortar made with CEM III/A 42.5 N cement. The red dot shows the value of the non-carbonated mortar, whereas the blue dot presents the activity concentrations measured after the mortar carbonation process at different curing ages.

The results obtained for the activity concentrations of ^235^U and ^210^Pb in CEM III/A 42.5 N indicated that the dataset does not conform to the normal distribution since the values calculated for the kurtosis, standardised bias, and Shapiro–Wilk test statistic for normality did not lie between −2 and +2. Furthermore, they were higher than the selected significance level (α = 0.05). The concentration distributions shown in Figure 2 are represented by box-and-whisker plots (Figures S1.1–S3.12). Box-and-whisker plots for ^235^U and ^210^Pb in CEM III/A 42.5 N are presented in Figure 3.

### 3.4. Comparison of Activity Concentrations of Radionuclides Belonging to the Natural Radioactive Series

**Figure 4:**
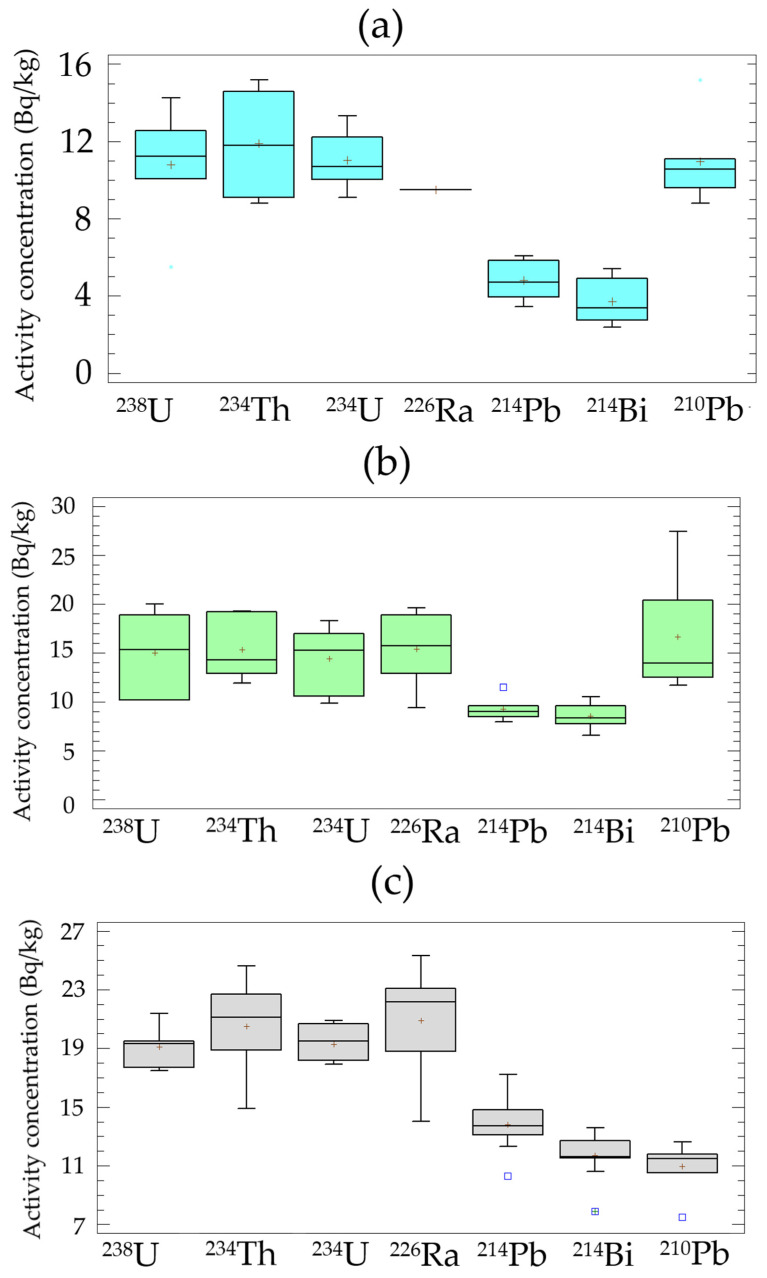
Distributions of the activity concentrations of ^238^U, ^234^Th, ^234^U, ^226^Ra, ^214^Pb, ^214^Bi, and ^210^Pb: (**a**) mortar made with CEM I 52.5 R-SR 3 cement, (**b**) mortar made with CEM II/A-S 42.5 N cement, and (**c**) mortar made with CEM III/A 42.5 N cement.

Figure 4 shows the distributions of the activity concentrations of the radionuclides belonging to the natural radioactive series of uranium (^238^U, ^234^Th, ^234^U, ^226^Ra, ^214^Pb, ^214^Bi, and ^210^Pb) for the 3 tested mortars. The activity concentrations correspond to the carbonated ground mortars. On the other hand, the distributions of the radioactive series of thorium for these mortars are presented in Supplementary S4. The box-and-whisker plots corroborate the results obtained in the normal distribution fit parameters (Figure 2).

### 3.5. Values Related to the Radiological Protection of the Tested Mortars

Table 4 presents the mean values of the activity concentration of ^226^Ra, ^214^Pb, ^212^Pb, ^40^K, absorbed (${\dot{D}}_{\gamma }$), and effective (E) doses for the standard room model (see Section 2.5) and ^222^Rn emanation rate. The results show an increase in several radiological parameters as the ground granulated blast-furnace slag content in the Portland cement used to manufacture the mortars increases.

## 4. Discussion

The obtained results confirmed our hypothesis that the carbonation process in mortars made with Portland cements without additions or made with ground granulated blast-furnace slag does not affect their chemical and physical properties.

The activity concentrations measured in 2 of the 3 tested mortars (Table 1, Table 2 and Table 3) were compared with the theoretical values determined from those of the individual anhydrous materials obtained in previous studies [44,45]. In the case of the mortar made with cement CEM I 52.5 R-SR 3, the calculated activity concentrations were 12.31 ± 2.5 Bq kg^−1^ for ^226^Ra, 7.2 ± 1.0 Bq kg^−1^ for ^232^Th, and 124.6 ± 6.2 Bq kg^−1^ for ^40^K. The calculated activity concentrations for the mortar made with the CEM II/A-S 42.5 N cement were 17.2 ± 3.2 Bq kg^−1^ for ^226^Ra, 9.5 ± 1.1 Bq kg^−1^ for ^232^Th, and 122.2 ± 6.1 Bq kg^−1^ for ^40^K. In the case of the CEM III/A 42.5 N cement, no activity concentrations were available to calculate the activity concentration. The activity concentrations of the solid and powdered carbonated mortars were statistically comparable (Figure 1). These results indicate that the analysis of the powdered mortars will allow us to obtain information about possible changes in the chemical or physical composition caused by the carbonation process. This finding made it possible to ensure that the radiochemical separations of isotopic uranium in the powdered samples using a 0.5 g aliquot were reproducible. The only 2 values that were significantly different on the mean were ^214^Bi and ^208^Tl in CEM II/A-S 42.5 N. However, the differences should not be considered as influencing the chemical composition since the same behaviour was not observed for ^226^Ra or ^214^Pb in the case of ^214^Bi, or ^228^Ac or ^212^Pb in the case of ^208^Tl. In the case of the variance used to compare the degree of dispersion, a significant difference was only observed for ^212^Pb, also in the CEM II/A-S 42.5 N cement. These observations ensure that the results obtained with the powdered mortar are consistent with the provisions of the European regulation, according to which it is necessary to determine the activity concentrations in the final construction material [2].

The carbonation process does not modify the chemical properties of the radionuclides belonging to the natural radioactive series or ^40^K of the 3 tested mortars (Figure 2). The activity concentrations of the natural radionuclides studied in the carbonated and non-carbonated samples followed a normal distribution, so there are no significant differences between them. Only the values of kurtosis and standard bias for the activity concentrations of ^210^Pb and ^235^U for the CEM III/A 42.5 N cement were outside of the range of −2 and +2, which would indicate significant deviations from a normal distribution. The Shapiro–Wilk test examines if a variable is normally distributed in a population, and the values obtained from the Shapiro–Wilk normality test for ^210^Pb and ^235^U were also lower than the selected significance level (α = 0.05), which indicates that this set of data does not belong to the normal distribution. Figure 3 shows that, in the case of ^235^U, the activity concentrations of the non-carbonated mortar made with CEM III/A 42.5 N have a marked positive bias. However, the rest of the activity concentrations would be distributed around the median of the values. In the case of ^210^Pb for the same mortar, it was observed that the activity concentration with a curing time of 3 days under water had a pronounced negative bias. Nevertheless, it was observed again that the remaining values were distributed around the median. In the case of uranium, the presence of calcite enhances U(VI) adsorption and release processes. The existence of dissolved Ca^2+^ and carbonate from the calcite would be responsible for the formation of the Ca_2_UO_2_(CO_3_)_3_^0^ (ac) complex that would promote the release of U(VI) from the surface of the solids [46]. The formation of these calcium uranyl carbonate complexes is higher at an alkaline pH, which would be those of the carbonation process of this study [47,48]. Likewise, the presence of Ca^2+^ alone prevents the adsorption of U(VI) on different surfaces, such as quartz, which is similar to the siliceous aggregate used to manufacture mortars [49]. However, our results did not show a decrease of uranium activity concentration (^238^U, ^235^U, and ^234^U). Therefore, the CaCO_3_ formed during the carbonation process would not produce the complexes indicated above, or the uranium would be part of more stable compounds that would inhibit these release processes. The observed decrease of ^210^Pb in the CEM III/A 42.5 N mortar can be attributed to the fact that the temperature for blast-furnace slag formation in the kiln is about 1500 °C [50]; accordingly, Pb would be partly lost by volatilization. The higher blast-furnace slag content in the CEM III/A 42.5 N, the more pronounced the effect. By contrast, this effect is not noticeable in the other two mortars with lower amounts of blast-furnace slag in the mix.

Carbonation would not influence the emanation of ^222^Rn from the tested mortars. The carbonation process produces some microstructural changes, i.e., a pore-size redistribution in the material, which increases or decreases depending on the type of cement [12]. Our results show that ^222^Rn emanation (activity concentrations of ^214^Pb and ^214^Bi) has performed the same in the 3 tested mortars (Figure 4). This statement is based on the fact that ^214^Pb and ^214^Pb concentrations were neither observed as being different between the solid and ground mortar, as mentioned above, nor between the carbonated and non-carbonated powdered samples (Table 1, Table 2, Table 3 and Table 4). The identified decrease of ^222^Rn through its offspring (^214^Pb and ^214^Bi) could not be due to radon losses as a consequence of the quality of the material source container [51] or incorrect sealing [52] since that had been verified in previous studies. Activity concentrations of the ^222^Rn parent, i.e., ^226^Ra, in the above-mentioned studies were higher [53]. However, the powdery sample did not fill the entire container in some samples, which could lead to a decrease in the determination of ^222^Rn due to a change in the counting geometry [54]. The results obtained in the distributions with full or partially filled containers were statistically indistinguishable. Neither the influence of particle size nor moisture on ^222^Rn emanation can be considered significant because all the samples were sieved at a particle size of 250 µm and dried at 105 °C at constant weight [55,56]. Therefore, the emanation rate (ɛ) observed in this study could be due to both the presence of the ground granulated blast-furnace slag and the cement clinker composition itself. In the case of the ground granulated blast-furnace slag, the emanation rate is 0.6 ± 0.1% [57], and in the case of the Portland cement, it is around 1% [58]; in neither case do they form any part of the pastes or mortars. 

On the other hand, the siliceous aggregate used to manufacture the mortars had an emanation of 6%; by contrast, the concentration of ^222^Rn (determined from ^214^Pb and ^214^Bi) was 4 Bq kg^−1^; accordingly, emanation can be considered negligible [18,59]. Emanation rates were inversely proportional to the ground granulated blast-furnace slag content (Table 4) and, therefore, the emanation would be due to the hydration reactions of the Portland cement clinker constituents since clinker is partially replaced by ground granulated blast-furnace slag in CEM II/A-S 42.5 N and CEM III/A 42.5 N cements [60]. The above-mentioned hydration reactions could cause variations in the ^226^Ra distribution on the particle surface and could therefore increase the ^222^Rn emanation [61]. The emanation rates found in this study are equivalent to those reported by Pakou et al. for the same type of materials [39]. These findings should be contrasted with direct measurements of ^222^Rn exhaled from the material, rather than with the emanation rate estimated by gamma spectrometry used in this work. However, as was the case in the activity concentrations determined by gamma spectrometry, no comparable results were found in the carbonated mortar samples. The effective dose due to gamma radiation was not modified by the carbonation process. The annual effective dose rates (E) shown in Table 4 increase proportionally with the amount of ground granulated blast-furnace slag since its contribution is higher than that of the Portland cement clinker [44]. On the other hand, the three mortars would be suitable, from the radiation protection point of view, since they are equivalent to the natural background values (0.24 mSv year^−1^, considering an absorbed dose of 50 nGy h^−1^).

## 5. Conclusions

The results of this study show that the carbonation process does not influence the radiological content of the tested mortars. First, it was found that the activity concentrations of the mortars were statistically indistinguishable from those calculated with the anhydrous materials. Mean and variance values obtained for the activity concentrations of ^234^Th, ^226^Ra, ^210^Pb, ^212^Pb, ^228^Ac, ^208^Tl, and ^40^K in both the hardened and powdered mortar prisms indicated that there were no significant differences in accuracy and precision. This fact was significant in verifying that the use of the radiochemical method of isotopic uranium separation (^238^U, ^235^U, and ^234^U) was possible. This type of analysis is more sensitive, but due to the fact that it requires fewer samples, it is necessary so as to confirm the sampling homogeneity.

The activity concentrations of ^238^U, ^235^U, and ^234^U were statistically comparable between the non-carbonated and carbonated mortars. This finding allows us to conclude that carbonation did not affect the chemical composition of the mortars since, in the case of an interaction between uranium and carbonate, significant losses of this radionuclide would occur.

The activity concentration results obtained in the ground samples showed that the first part of the natural radioactive chain of uranium (^238^U, ^234^Th, ^234^U, and ^226^Ra) was in equilibrium, while the final part of the chain (^214^Pb, ^214^Bi, and ^210^Pb) was not, as their activity concentrations were lower. In the case of ^210^Pb, the higher presence of ground granulated blast-furnace slag would justify this decrease in the activity concentration since blast-furnace slag is generated at temperatures close to 1500 °C; therefore, ^210^Pb is partially removed by volatilisation. 

The ^222^Rn emanation rates indicated that the hydration reactions of the Portland cement are responsible for this decrease, and not the mortar pore-size re-distribution, which can occur via the carbonation process. Finally, as a consequence of the above findings, the annual effective dose rates due to the external gamma radiation for the 3 mortars were found to be equivalent to the environmental background (0.24 mSv).

The novelty of this research program relies mainly on the fact that it studied for the first time the radiological characteristics of carbonated Portland cement mortars made with ground granulated blast-furnace slag. 

Our results allow us to conclude that, under the conditions of our study, the carbonation process did not cause changes in the chemical composition or in the physical structure due to a possible mortar pore redistribution. On the other hand, this research will provide new insights into the low radiological risk from carbonated cement-based materials. Through this research, the community will further realize the promotion of the use of cement-based materials, of which carbonates along the time. Civil engineers may also consider mortars and concretes as harmless building materials. Moreover, the assessment performed in this study will convey valuable information for future research that will explore the activity concentration of building materials containing other NORM materials. 

## Figures and Tables

**Table 1:** Activity concentrations of the prisms of hardened mortars (The prisms were ground to measure the activity concentrations): (a) non-carbonated mortars and (b) carbonated mortars at different curing ages (0, 1, 3, 7, 14, and 28 days).

Cement	Days	Uranium Series					^235^U	Thorium Series			^40^K
		^234^Th	^226^Ra	^214^Pb	^214^Bi	^210^Pb		^228^Ac	^212^Pb	^208^Tl	
C1	-	9.9 ± 2.6	13.9 ± 3.8	4.81 ± 0.53	4.42 ± 0.35	<8.2	<2.7	6.43 ± 0.40	7.58 ± 0.63	3.42 ± 0.48	108.2 ± 6.3
		14.8 ± 4.1	15.4 ± 6.0	6.39 ± 0.80	6.11 ± 0.67	<11.1	<4.8	5.51 ± 0.78	7.21 ± 0.65	3.22 ± 0.38	114.2 ± 6.7
		8.7 ± 1.2	<7.9	6.02 ± 0.54	6.13 ± 0.38	7.4 ± 1.4	<1.8	5.41 ± 0.38	6.31 ± 0.53	3.24 ± 0.26	106.7 ± 5.3
	0	8.5 ± 2.6	<16.1	5.56 ± 0.81	4.43 ± 0.70	<9.9	<4.9	6.40 ± 0.90	6.85 ± 0.62	2.64 ± 0.38	122.5 ± 7.3
	1	8.7 ± 2.1	11.5 ± 1.3	5.04 ± 0.45	5.13 ± 0.33	7.3 ± 2.4	<1.5	4.62 ± 0.34	4.54 ± 0.39	1.93 ± 0.18	71.3 ± 3.8
	3	<7.9	9.0 ± 2.7	5.42 ± 0.83	3.70 ± 0.54	<9.0	<2.6	4.98 ± 0.45	6.94 ± 0.58	3.00 ± 0.23	111.1 ± 6.6
	7	<12.2	<20.4	7.15 ± 0.90	8.5 ± 1.0	<11.1	<6.2	7.2 ± 1.3	7.76 ± 0.38	3.97 ± 0.61	126.2 ± 7.2
	14	9.9 ± 3.5	<11.0	6.0 ± 1.0	4.22 ± 0.63	<8.8	<3.1	5.02 ± 0.51	7.13 ± 0.81	3.33 ± 0.26	107.0 ± 7.2
	28	12.9 ± 2.8	6.1 ± 3.0	6.23 ± 0.56	6.09 ± 0.40	<5.1	<1.8	5.49 ± 0.39	6.26 ± 0.53	2.83 ± 0.24	96.6 ± 5.0
C2	-	13.2 ± 2.4	11.9 ± 4.1	6.75 ± 0.61	6.29 ± 0.58	<6.1	<1.9	5.95 ± 0.37	7.94 ± 0.65	3.91 ± 0.38	98.2 ± 5.2
		11.6 ± 3.2	27.1 ± 8.3	6.50 ± 0.85	6.32 ± 0.68	11.8 ± 4.2	<5.0	7.00 ± 0.87	7.87 ± 0.70	3.59 ± 0.42	100.8 ± 6.5
		11.9 ± 2.8	8.4 ± 2.6	7.12 ± 0.61	5.72 ± 0.40	<6.4	<1.8	6.03 ± 0.41	7.27 ± 0.61	3.19 ± 0.26	93.4 ± 4.8
	0	10.9 ± 3.2	17.0 ± 3.4	8.05 ± 0.76	6.74 ± 0.79	<10.0	<3.0	6.71 ± 0.39	8.13 ± 0.89	3.37 ± 0.26	120.0 ± 7.3
	1	17.2 ± 3.5	<14.3	9.34 ± 0.90	8.51 ± 0.66	13.4 ± 3.7	<3.8	6.94 ± 0.62	7.00 ± 0.61	3.33 ± 0.35	120.6 ± 6.5
	3	14.0 ± 2.2	<10.0	7.67 ± 0.67	7.83 ± 0.46	<6.1	<2.0	7.05 ± 0.45	7.20 ± 0.61	3.52 ± 0.28	106.5 ± 5.5
	7	14.4 ± 2.4	8.4 ± 2.1	8.77 ± 0.71	9.59 ± 0.43	10.9 ± 2.3	<1.4	6.50 ± 0.36	7.22 ± 0.60	3.24 ± 0.23	89.2 ± 4.4
	14	13.0 ± 3.6	19.0 ± 5.6	7.29 ± 0.94	5.98 ± 0.50	11.8 ± 3.7	<2.9	6.44 ± 0.53	8.3 ± 3.3	3.94 ± 0.57	104.6 ± 5.9
	28	15.1 ± 3.4	16.4 ± 7.9	5.98 ± 0.83	6.05 ± 0.75	<11.1	<5.2	7.6 ± 1.0	7.66 ± 0.69	3.93 ± 0.47	95.9 ± 6.6
C3	-	15.9 ± 2.7	19.3 ± 3.0	15.1 ± 1.2	14.50 ± 0.75	<7.8	<2.6	8.18 ± 0.53	10.7 ± 1.0	4.94 ± 0.55	99.1 ± 5.8
		16.6 ± 3.1	21.7 ± 7.3	15.0 ± 1.4	14.6 ± 1.0	8.8 ± 3.4	<5.0	8.66 ± 0.86	9.76 ± 0.84	4.69 ± 0.55	97.8 ± 6.5
		12.9 ± 2.0	15.9 ± 2.9	12.4 ± 1.0	12.90 ± 0.52	<4.6	<1.4	8.77 ± 0.41	8.87 ± 0.73	4.05 ± 0.28	85.3 ± 4.1
	0	22.7 ± 4.2	21.7 ± 8.0	11.5 ± 1.2	11.2 ± 1.0	<10.8	<6.0	11.1 ± 1.1	11.6 ± 1.0	5.17 ± 0.61	133.1 ± 8.4
	1	18.0 ± 3.5	19.9 ± 6.7	11.8 ± 1.3	9.45 ± 0.64	<8.9	<2.9	7.92 ± 0.43	9.78 ± 0.93	4.37 ± 0.31	116.0 ± 7.1
	3	18.9 ± 3.3	12.9 ± 3.2	11.7 ± 1.0	10.20 ± 0.63	<7.0	<2.0	7.85 ± 0.45	8.78 ± 0.74	3.73 ± 0.30	111.3 ± 5.6
	7	17.7 ± 3.0	21.1 ± 5.6	14.6 ± 1.4	13.67 ± 0.93	10.7 ± 3.3	<2.4	7.74 ± 0.48	9.98 ± 0.89	4.64 ± 0.50	109.6 ± 6.1
	14	16.9 ± 2.3	14.0 ± 2.4	12.7 ± 1.0	12.97 ± 0.55	<5.1	<1.8	7.53 ± 0.40	8.44 ± 0.70	3.82 ± 0.27	113.7 ± 5.3
	28	21.4 ± 3.5	<13.3	13.4 ± 1.2	11.85 ± 0.80	11.0 ± 4.0	<4.0	8.80 ± 0.70	8.88 ± 0.76	3.59 ± 0.40	99.8 ± 5.9

The uncertainties cited are for a coverage factor of *k* = 2.

**Table 2:** Activity concentrations of the prisms of hardened mortars: (a) non-carbonated mortars and (b) carbonated mortars at different curing ages (0, 1, 3, 7, 14, and 28 days).

Cement	Days	Uranium Series					^235^U	Thorium Series			^40^K
		^234^Th	^226^Ra	^214^Pb	^214^Bi	^210^Pb		^228^Ac	^212^Pb	^208^Tl	
C1	-	10.7 ± 1.9	11.9 ± 2.1	6.67 ± 0.71	5.33 ± 0.37	14.0 ± 2.7	<1.7	8.11 ± 0.70	7.88 ± 0.69	4.06 ± 0.42	124.2 ± 5.5
		9.4 ± 2.2	13.3 ± 4.2	8.02 ± 0.79	6.89 ± 0.55	9.5 ± 2.5	<3.4	6.91 ± 0.60	7.57 ± 0.65	4.04 ± 0.41	112.7 ± 6.0
		11.7 ± 2.3	9.4 ± 2.3	6.73 ± 0.58	5.90 ± 0.40	12.0 ± 2.9	<2.2	6.39 ± 0.41	6.73 ± 0.57	3.53 ± 0.28	98.4 ± 5.1
	0	13.7 ± 4.2	<17.9	3.45 ± 0.72	2.39 ± 0.65	15.2 ± 4.5	<5.5	4.1 ± 1.1	6.56 ± 0.62	2.72 ± 0.46	104.0 ± 7.1
	1	8.8 ± 1.8	9.5 ± 2.6	6.08 ± 0.52	5.40 ± 0.36	9.6 ± 2.1	<1.3	6.78 ± 0.37	6.49 ± 0.54	3.21 ± 0.24	113.4 ± 5.4
	3	14.6 ± 3.3	<9.5	3.95 ± 0.51	3.57 ± 0.66	8.8 ± 2.0	<2.8	5.88 ± 0.52	6.99 ± 0.70	4.04 ± 0.58	125.4 ± 7.6
	7	15.2 ± 4.2	<18.8	5.19 ± 0.91	3.21 ± 0.74	10.2 ± 3.4	<5.7	5.8 ± 1.0	7.89 ± 0.72	3.21 ± 0.55	110.7 ± 7.6
	14	9.9 ± 2.3	<10.0	4.25 ± 0.85	2.75 ± 0.37	10.9 ± 3.1	<2.7	4.98 ± 0.50	7.72 ± 0.65	3.83 ± 0.58	121.6 ± 7.5
	28	9.1 ± 2.1	<9.9	5.83 ± 0.55	4.91 ± 0.42	11.1 ± 3.0	<1.9	6.56 ± 0.47	6.80 ± 0.58	3.60 ± 0.30	107.7 ± 5.8
C2	-	7.9 ± 1.3	14.2 ± 1.7	9.03 ± 0.81	7.89 ± 0.53	5.7 ± 1.4	<1.2	5.36 ± 0.30	7.11 ± 0.61	3.69 ± 0.32	85.7 ± 4.4
		19.1 ± 4.0	<14.0	11.0 ± 1.1	8.84 ± 0.72	14.0 ± 3.3	<0.0	8.97 ± 0.73	9.22 ± 0.80	4.72 ± 0.49	108.2 ± 6.4
		12.7 ± 2.5	13.8 ± 3.6	9.81 ± 0.80	8.17 ± 0.48	6.8 ± 2.0	<1.6	7.28 ± 0.44	8.30 ± 0.69	4.17 ± 0.31	98.0 ± 5.0
	0	15.2 ± 3.0	17.1 ± 3.9	8.0 ± 1.0	6.59 ± 0.53	15.0 ± 3.8	<2.8	6.09 ± 0.54	9.32 ± 0.81	4.23 ± 0.31	127.8 ± 7.8
	1	19.2 ± 3.1	14.4 ± 5.2	9.6 ± 1.0	9.64 ± 0.73	11.7 ± 3.3	<4.3	6.03 ± 0.89	8.21 ± 0.71	3.95 ± 0.48	117.0 ± 6.7
	3	12.9 ± 2.6	9.4 ± 2.5	8.91 ± 0.76	8.96 ± 0.60	27.4 ± 4.5	<2.0	6.89 ± 0.48	7.69 ± 0.65	4.00 ± 0.32	118.5 ± 6.0
	7	13.3 ± 2.2	12.9 ± 2.6	11.52 ± 0.94	10.55 ± 0.67	20.4 ± 3.1	<1.4	6.34 ± 0.49	5.11 ± 0.46	4.01 ± 0.34	128.8 ± 6.1
	14	11.9 ± 2.8	19.6 ± 3.2	8.48 ± 0.78	7.80 ± 0.59	12.5 ± 3.6	<2.8	8.2 ± 1.0	9.7 ± 6.5	4.80 ± 0.34	113.0 ± 9.5
	28	19.3 ± 4.1	18.9 ± 7.1	9.1 ± 1.1	7.79 ± 0.88	12.9 ± 3.6	<5.7	6.2 ± 1.0	9.13 ± 0.81	4.13 ± 0.59	118.9 ± 7.8
C3	-	21.1 ± 3.8	25.3 ± 6.0	14.8 ± 1.8	11.61 ± 0.77	11.6 ± 2.9	<3.8	10.29 ± 0.58	11.6 ± 1.1	6.37 ± 0.55	105.8 ± 8.2
		23.3 ± 5.6	<24.4	10.3 ± 1.4	7.9 ± 1.1	<13.6	<8.3	10.3 ± 1.5	12.0 ± 1.1	5.76 ± 0.77	106.0 ± 9.1
		17.0 ± 2.0	21.7 ± 3.9	13.5 ± 1.1	11.54 ± 0.59	12.6 ± 2.8	<1.8	10.24 ± 0.54	11.29 ± 0.93	5.12 ± 0.38	98.0 ± 5.1
	0	20.4 ± 5.8	<23.5	17.2 ± 1.7	13.6 ± 1.3	<11.4	<7.4	8.7 ± 1.2	10.29 ± 0.94	4.64 ± 0.75	122.9 ± 9.1
	1	18.9 ± 3.5	18.8 ± 5.2	15.6 ± 1.5	13.30 ± 0.70	11.5 ± 3.8	<2.8	10.2 ± 1.1	9.9 ± 1.0	5.48 ± 0.56	127.0 ± 7.7
	3	14.9 ± 1.8	22.6 ± 4.3	13.9 ± 1.1	11.55 ± 0.66	7.5 ± 1.4	<2.1	7.40 ± 0.52	8.87 ± 0.75	4.37 ± 0.35	97.0 ± 5.4
	7	21.8 ± 3.7	23.1 ± 7.4	13.7 ± 1.5	10.6 ± 1.0	11.8 ± 2.4	<3.3	7.75 ± 0.65	11.1 ± 1.1	5.31 ± 0.75	114.4 ± 7.8
	14	24.6 ± 3.3	14.0 ± 4.4	13.1 ± 1.1	12.51 ± 0.67	11.3 ± 2.4	<2.9	7.40 ± 0.51	8.85 ± 0.75	4.65 ± 0.37	94.9 ± 5.2
	28	22.7 ± 4.7	<19.0	12.3 ± 1.3	12.7 ± 1.0	10.5 ± 3.8	<6.2	7.6 ± 1.1	10.39 ± 0.92	5.94 ± 0.71	106.4 ± 7.6

The uncertainties are quoted for a coverage factor of *k* = 2.

**Table 3:** Activity concentrations of ^238^U, ^235^U, and ^234^U of powdered mortars determined by radiochemical separation.

Cement	Days	^238^U	^235^U	^234^U
C1	-	11.62 ± 0.89	0.83 ± 0.20	12.01 ± 0.91
		13.45 ± 0.90	0.50 ± 0.15	13.43 ± 0.90
		14.0 ± 1.1	<0.7	12.8 ± 1.1
	0	5.5 ± 1.6	1.04 ± 0.72	9.1 ± 2.0
	1	11.85 ± 0.82	<0.6	11.32 ± 0.82
	3	10.07 ± 0.86	0.40 ± 0.17	10.09 ± 0.85
	7	12.57 ± 0.82	<0.5	12.22 ± 0.82
	14	14.25 ± 0.92	<0.6	13.31 ± 0.89
	28	10.60 ± 0.88	0.61 ± 0.23	10.03 ± 0.85
C2	-	7.1 ± 1.4	<1.1	8.7 ± 1.3
		15.9 ± 1.0	<0.5	17.6 ± 1.1
		17.1 ± 1.1	0.89 ± 0.20	16.0 ± 1.0
	0	15.5 ± 1.0	0.68 ± 0.23	16.8 ± 1.0
	1	18.9 ± 1.1	0.73 ± 0.18	18.3 ± 1.1
	3	10.2 ± 1.2	<1.0	10.6 ± 1.2
	7	15.17 ± 0.94	0.73 ± 0.22	13.79 ± 0.90
	14	10.2 ± 1.3	<1.4	9.9 ± 1.2
	28	20.0 ± 1.2	<0.5	17.0 ± 1.1
C3	-	17.6 ± 2.6	1.08 ± 0.28	17.9 ± 2.6
		17.7 ± 1.1	<0.7	17.9 ± 1.2
		21.4 ± 1.2	0.61 ± 0.23	20.7 ± 1.2
	0	19.3 ± 1.2	<0.7	18.2 ± 1.1
	1	19.3 ± 1.2	<0.9	20.7 ± 1.3
	3	17.5 ± 1.2	0.64 ± 0.24	19.5 ± 1.2
	7	20.8 ± 1.2	0.72 ± 0.22	19.5 ± 1.1
	14	18.9 ± 1.1	0.61 ± 0.18	20.9 ± 1.2
	28	19.5 ± 1.1	0.69 ± 0.22	18.2 ± 1.1

The uncertainties are quoted for a coverage factor of *k* = 2.

**Table 4:** Activity concentrations of ^226^Ra, ^214^Pb, ^212^Pb, ^40^K, absorbed dose (${\dot{D}}_{\gamma }$), effective dose (E), and ^222^Rn emanation rate for the three tested mortars.

Parameter	Cement Type Used to Make the Mortar		
	CEM I 52.5 R-SR 3	CEM II/A-S 42.5 N	CEM III/A 42.5 N
^226^Ra (Bq kg^−1^)	9.5 ± 2.6	16.1 ± 2.9	20.1 ± 3.1
^214^Pb (Bq kg^−1^)	4.73 ± 0.85	9.3 ± 1.0	14.5 ± 1.6
^212^Pb (Bq kg^−1^)	7.11 ± 0.50	9.2 ± 1.0	10.01 ± 0.73
^40^K (Bq kg^−1^)	114.2 ± 7.0	120.2 ± 5.3	112 ± 11
${\dot{D}}_{\gamma }$ (nGy h^−1^)	25.7 ± 3.3	34.5 ± 3.8	38.5 ± 4.0
E (mSv y^−1^)	0.126 ± 0.016	0.169 ± 0.019	0.189 ± 0.020
ɛ	0.50 ± 0.21	0.42 ± 0.12	0.279 ± 0.068

Uncertainties are quoted for a coverage factor of *k* = 2.

## Data Availability

The data presented in this study are available upon request from the corresponding author.

## Supplementary Materials

The following supporting information can be downloaded at: https://www.mdpi.com/article/10.3390/ma15093395/s1.
