# Supplementary material for: Radiological Characteristics of Carbonated Portland Cement Mortars Made with GGBFS
Source: Materials (Basel). 2022 May 9;15(9):3395. doi: 10.3390/ma15093395 (PMC9100595; doi:10.3390/ma15093395)
Supplement: Supplementary file 1 [file materials-15-03395-s001.zip › materials-1687538-supplementary.pdf]

*materials*

MDPI

*Article*

# Supplementary Information

**Supplementary S1. Box-and-whisker and scatter plots for the radionuclides of mortars made with CEM I 52.5 R-SR 3 cement.**

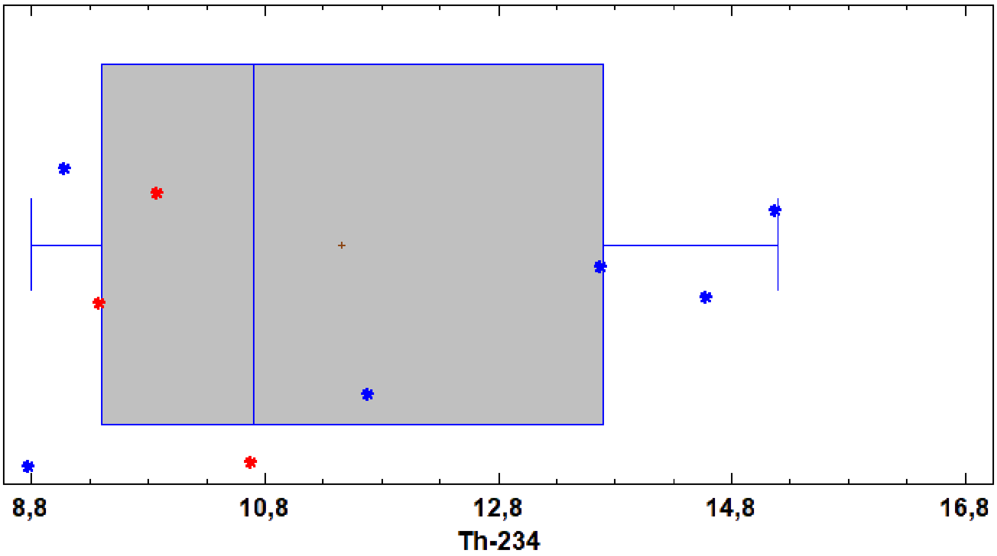


**Figure S1.1.** $^{234}Th$ activity concentrations box-and-whisker plot for ground mortar made with CEM I 52.5 R-SR 3 cement. The red dot shows the value for non-carbonated mortar and the blue dot shows the activity concentrations at different curing ages.

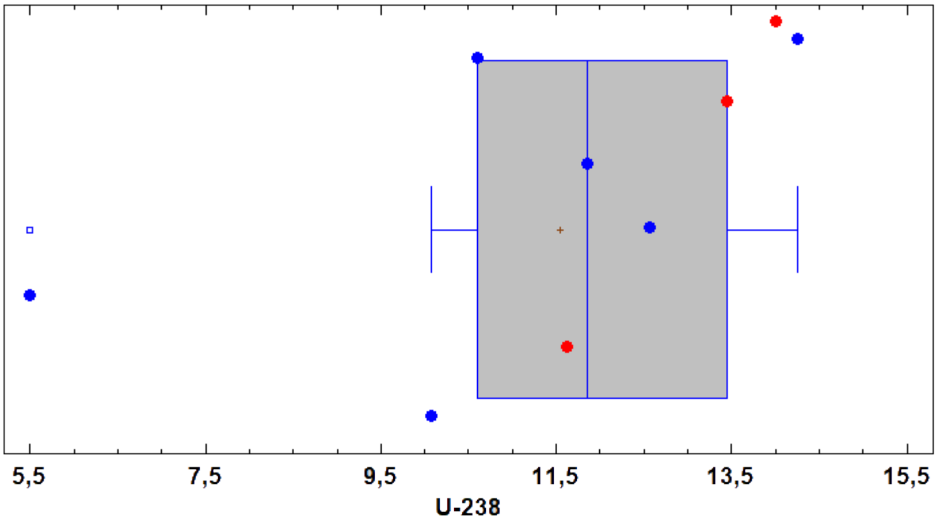


**Figure S1.2**. $^{238}U$ activity concentrations box-and-whisker plot for ground mortar made with CEM I 52.5 R-SR 3 cement. The red dot shows the value for non-carbonated mortar and the blue dot shows the activity concentrations at different curing ages.

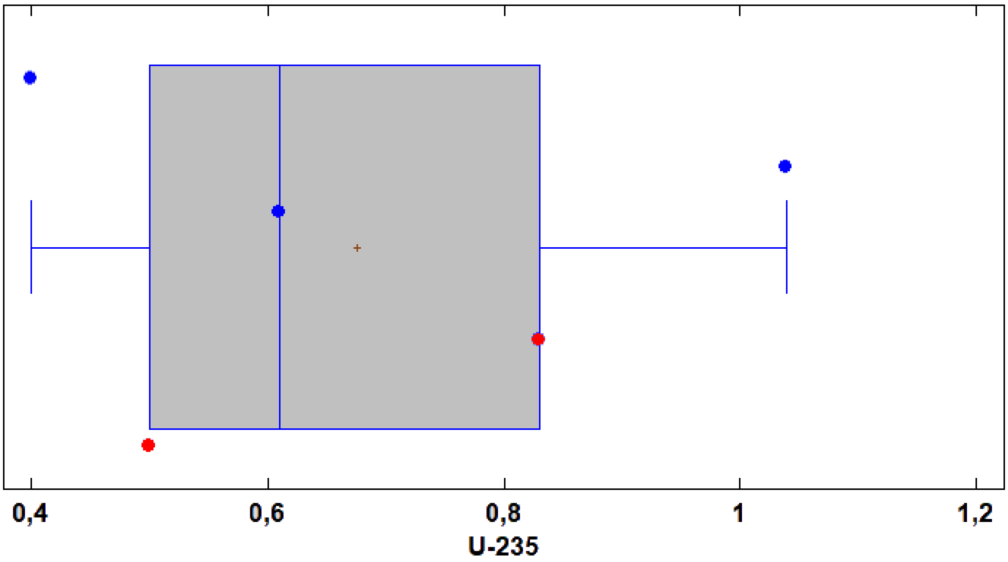


**Figure S1.3**. $^{235}U$ activity concentrations box-and-whisker plot for ground mortar made with CEM I 52.5 R-SR 3 cement. The red dot shows the value for non-carbonated mortar and the blue dot shows the activity concentrations at different curing ages.

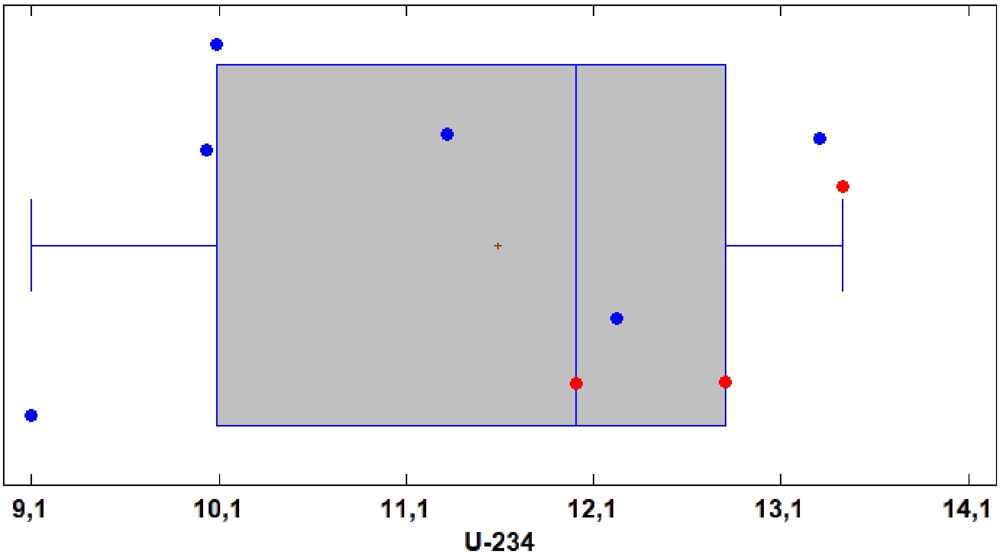


**Figure S1.4**. $^{234}U$ activity concentrations box-and-whisker plot for ground mortar made with CEM I 52.5 R-SR 3 cement. The red dot shows the value for non-carbonated mortar and the blue dot shows the activity concentrations at different curing ages.

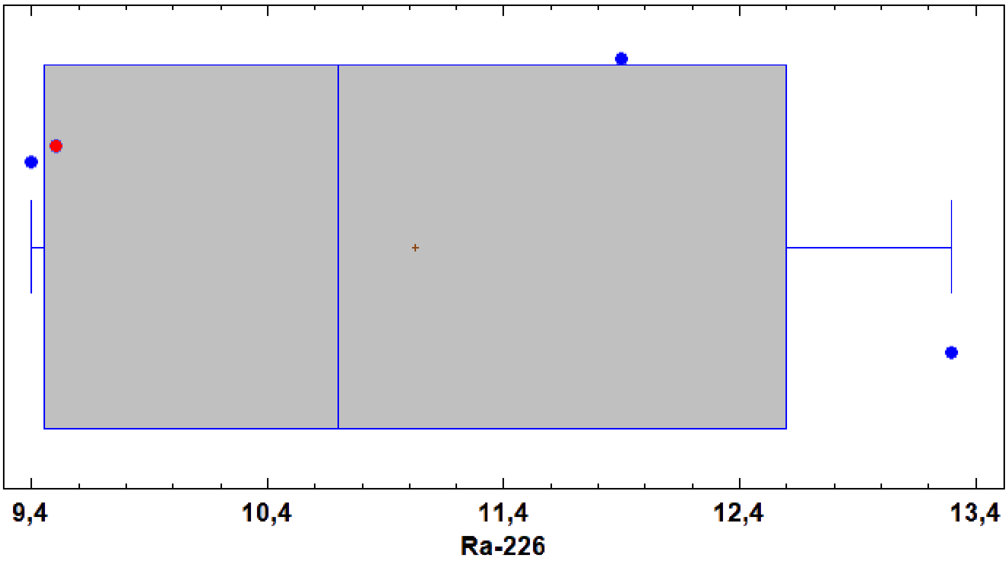


**Figure S1.5**. $^{226}Ra$ activity concentrations box-and-whisker plot for ground mortar made with CEM I 52.5 R-SR 3 cement. The red dot shows the value for non-carbonated mortar and the blue dot shows the activity concentrations at different curing ages.

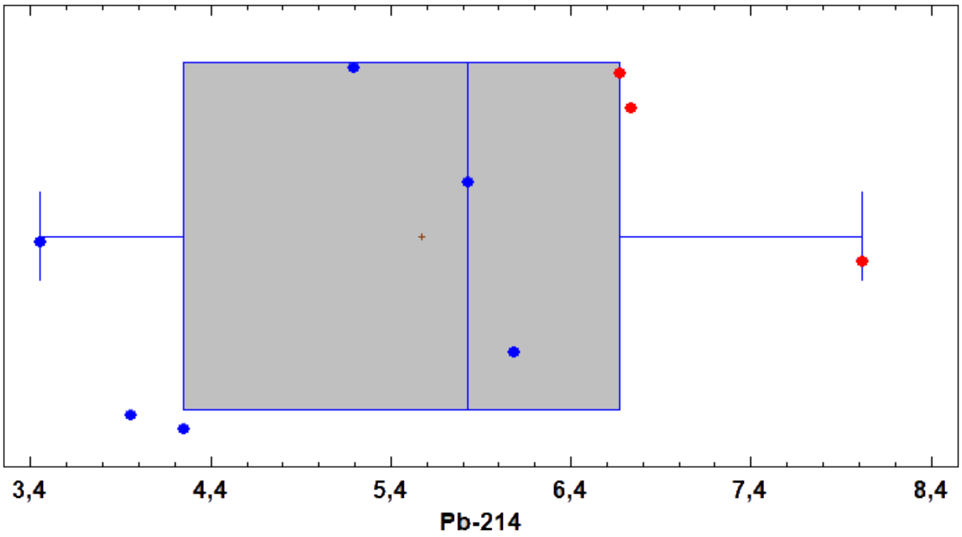


**Figure S1.6**. $^{214}Pb$ activity concentrations box-and-whisker plot for ground mortar made with CEM I 52.5 R-SR 3 cement. The red dot shows the value for non-carbonated mortar and the blue dot shows the activity concentrations at different curing ages.

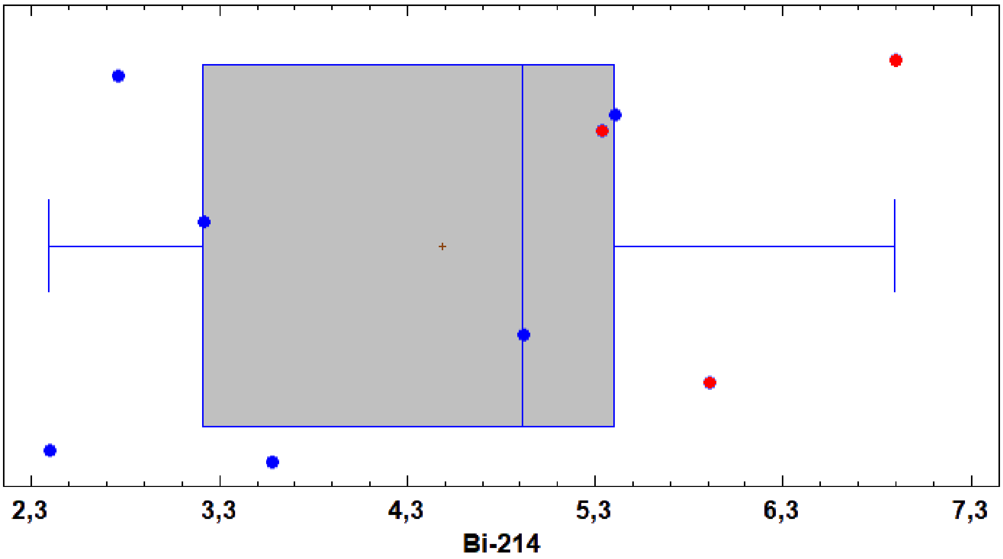


**Figure S1.7.** $^{214}$Bi activity concentrations box-and-whisker plot for ground mortar made with CEM I 52.5 R-SR 3 cement. The red dot shows the value for non-carbonated mortar and the blue dot shows the activity concentrations at different curing ages.

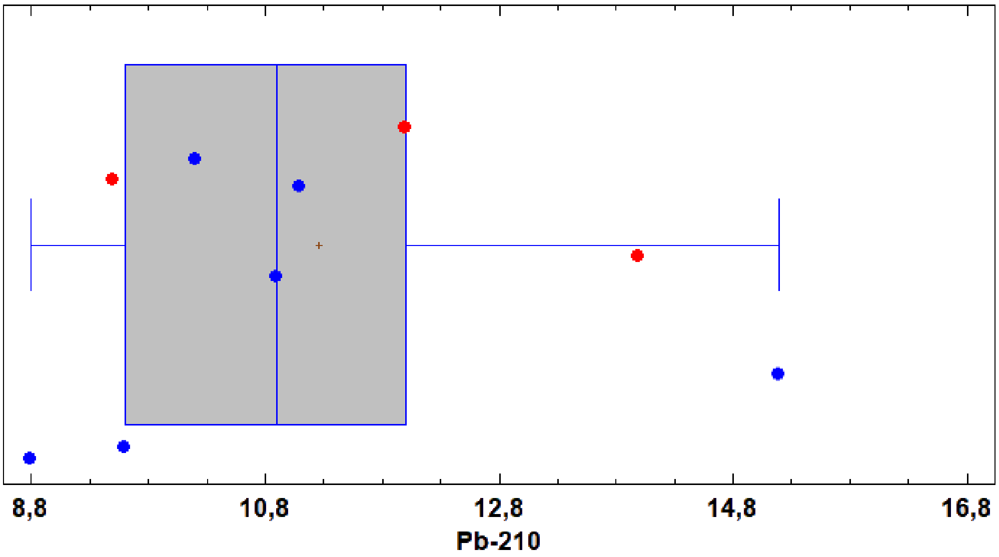


**Figure S1.8.** $^{210}$Pb activity concentrations box-and-whisker plot for ground mortar made with CEM I 52.5 R-SR 3 cement. The red dot shows the value for non-carbonated mortar and the blue dot shows the activity concentrations at different curing ages.

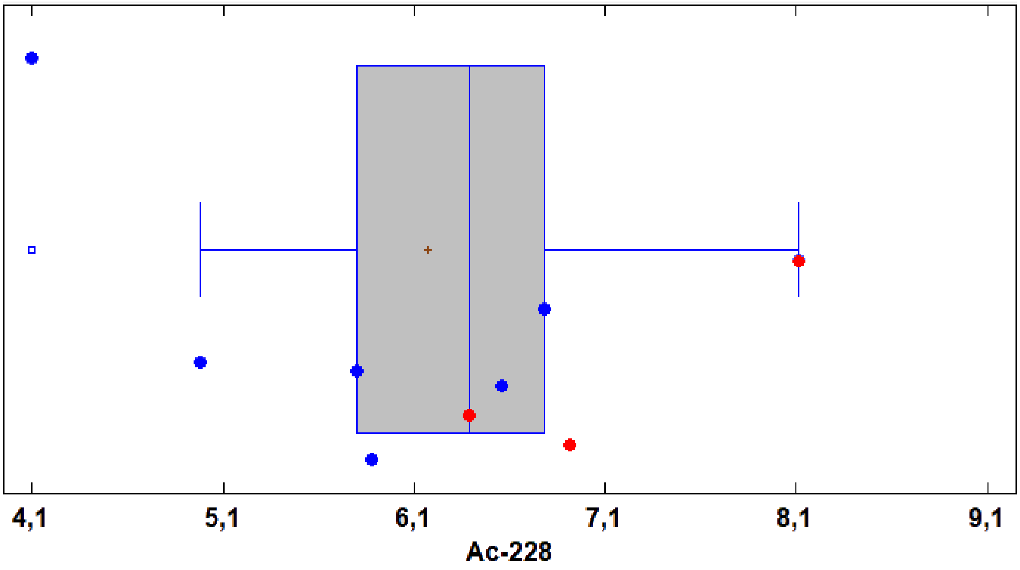


**Figure S1.9**. $^{228}$Ac activity concentrations box-and-whisker plot for ground mortar made with CEM I 52.5 R-SR 3 cement. The red dot shows the value for non-carbonated mortar and the blue dot shows the activity concentrations at different curing ages.

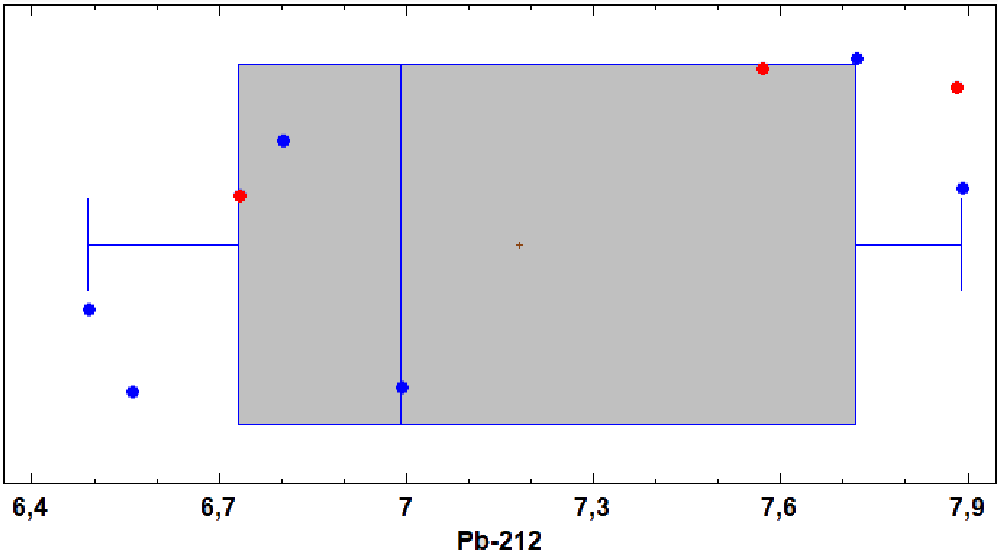


**Figure S1.10**. $^{212}$Pb activity concentrations box-and-whisker plot for ground mortar made with CEM I 52.5 R-SR 3 cement. The red dot shows the value for non-carbonated mortar and the blue dot shows the activity concentrations at different curing ages.

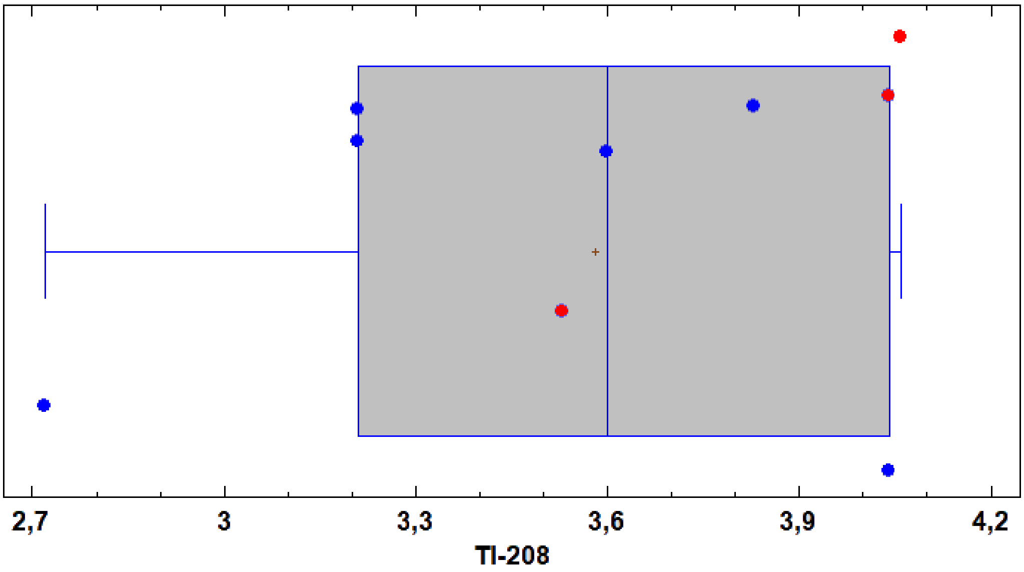


**Figure S1.11.** $^{212}$Pb activity concentrations box-and-whisker plot for ground mortar made with CEM I 52.5 R-SR 3 cement. The red dot shows the value for non-carbonated mortar and the blue dot shows the activity concentrations at different curing ages.

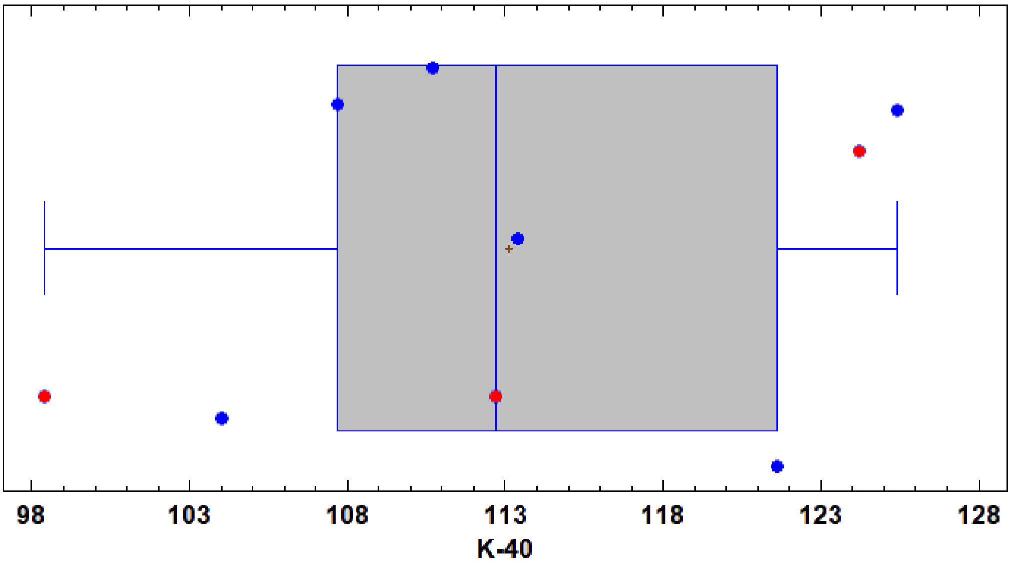


**Figure S1.12.** $^{40}$K activity concentrations box-and-whisker plot for ground mortar made with CEM I 52.5 R-SR 3 cement. The red dot shows the value for non-carbonated mortar and the blue dot shows the activity concentrations at different curing ages.

*materials* MDPI

**Supplementary S2. Box-and-whisker and scatter plots for the radionuclides of mortars made with CEM II/A-S 42.5N cement.**

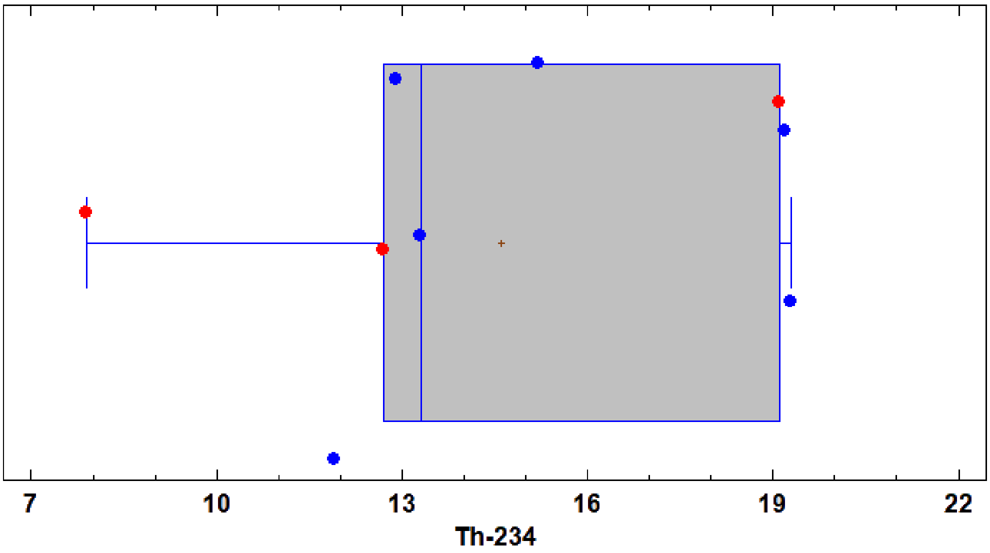


**Figure S2.1.** $^{234}$Th activity concentrations box-and-whisker plot for ground mortar made with CEM II/A-S 42.5 N cement. The red dot shows the value for non-carbonated mortar and the blue dot shows the activity concentrations at different curing ages.

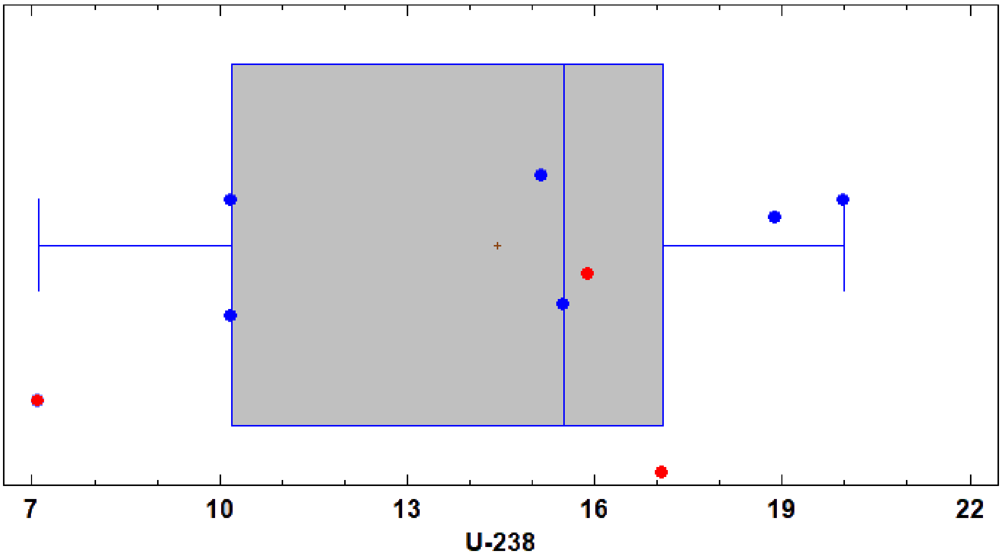


**Figure S2.2.** $^{238}$U activity concentrations box-and-whisker plot for ground mortar made with CEM II/A-S 42.5 N cement. The red dot shows the value for non-carbonated mortar and the blue dot shows the activity concentrations at different curing ages.

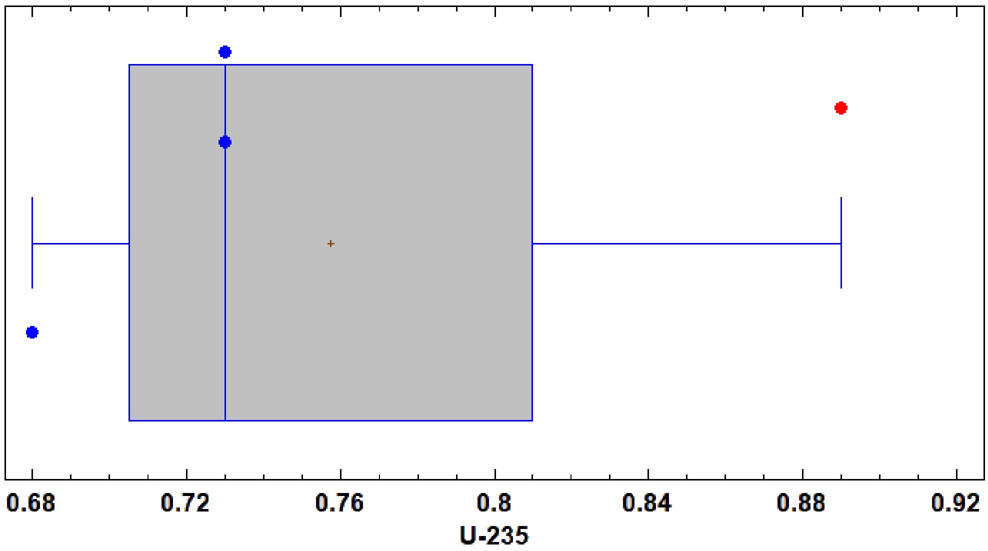


**Figure S2.3.** $^{235}U$ activity concentrations box-and-whisker plot for ground mortar made with CEM II/A-S 42.5 N cement. The red dot shows the value for non-carbonated mortar and the blue dot shows the activity concentrations at different curing ages.

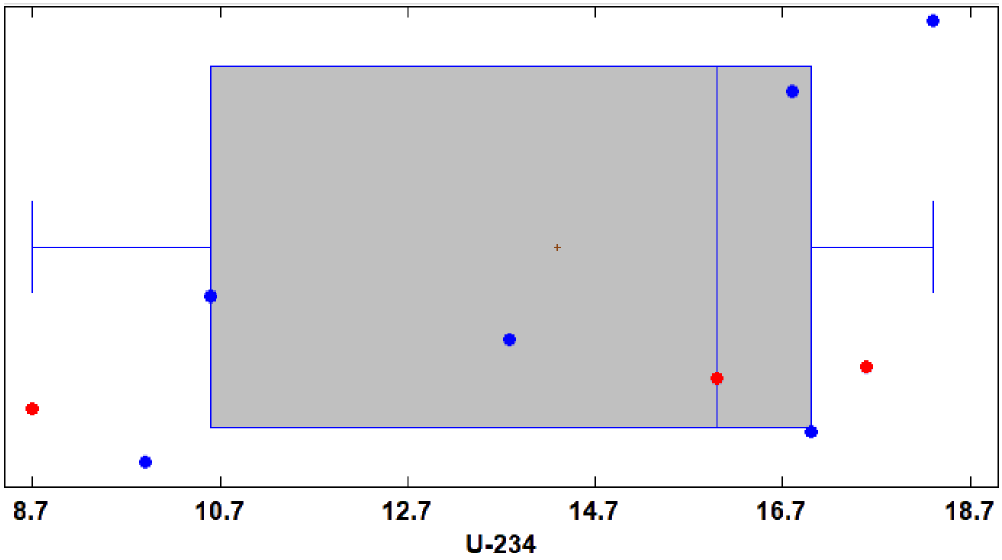


**Figure S2.4.** $^{234}U$ activity concentrations box-and-whisker plot for ground mortar made with CEM II/A-S 42.5 N cement. The red dot shows the value for non-carbonated mortar and the blue dot shows the activity concentrations at different curing ages.

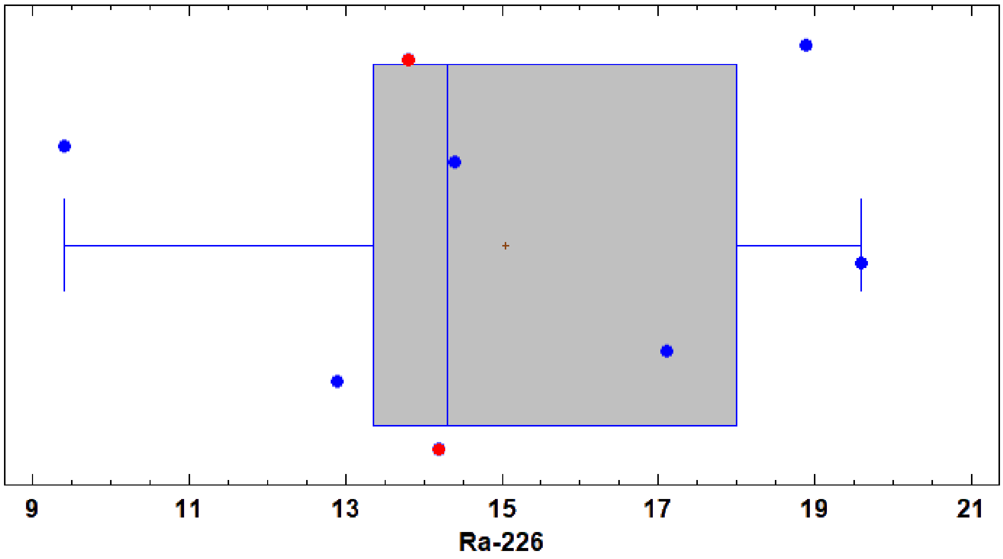


**Figure S2.5.** $^{226}$Ra activity concentrations box-and-whisker plot for ground mortar made with CEM II/A-S 42.5 N cement. The red dot shows the value for non-carbonated mortar and the blue dot shows the activity concentrations at different curing ages.

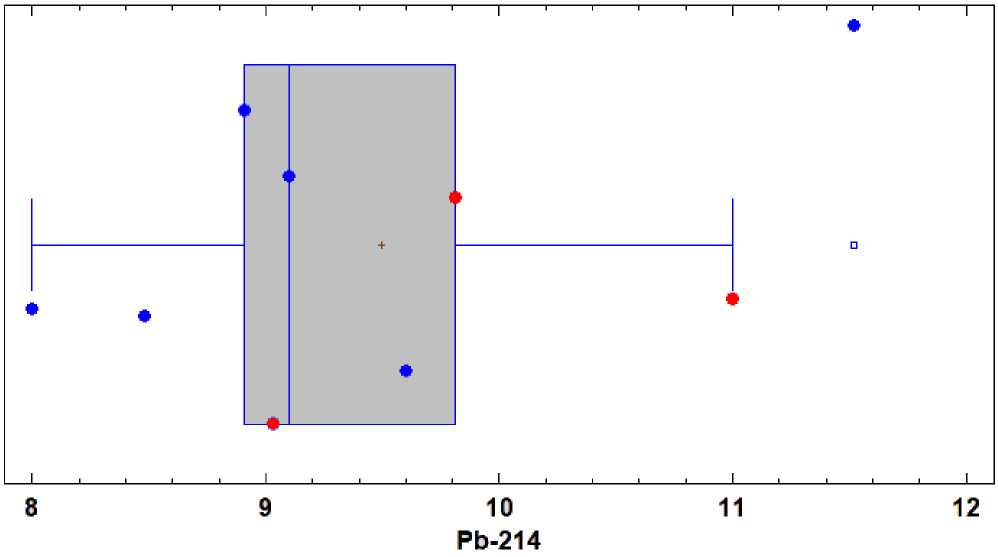


**Figure S2.6.** $^{214}$Pb activity concentrations box-and-whisker plot for ground mortar made with CEM II/A-S 42.5 N cement. The red dot shows the value for non-carbonated mortar and the blue dot shows the activity concentrations at different curing ages.

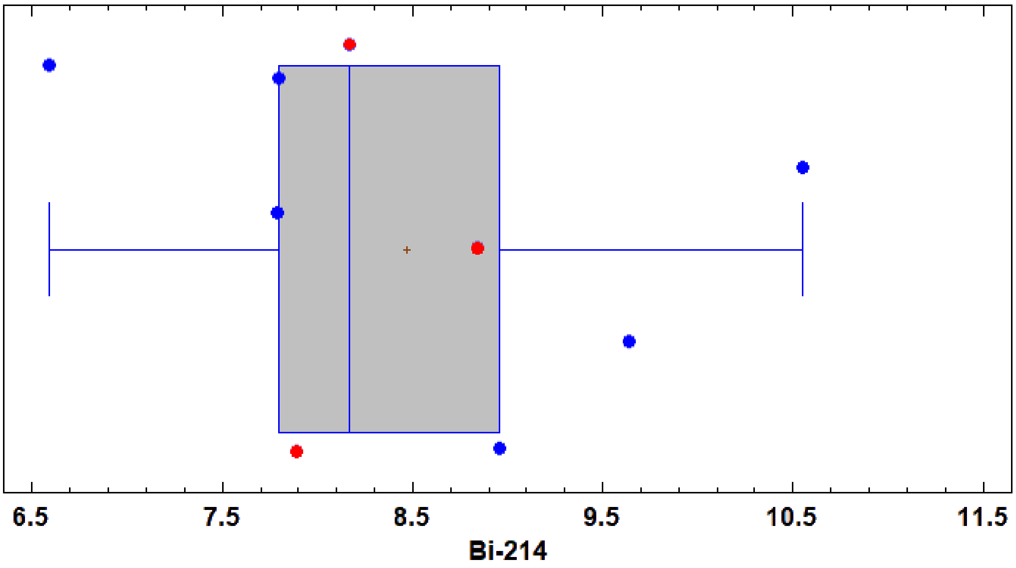


**Figure S2.7.** $^{214}$Bi activity concentrations box-and-whisker plot for ground mortar made with CEM II/A-S 42.5 N cement. The red dot shows the value for non-carbonated mortar and the blue dot shows the activity concentrations at different curing ages.

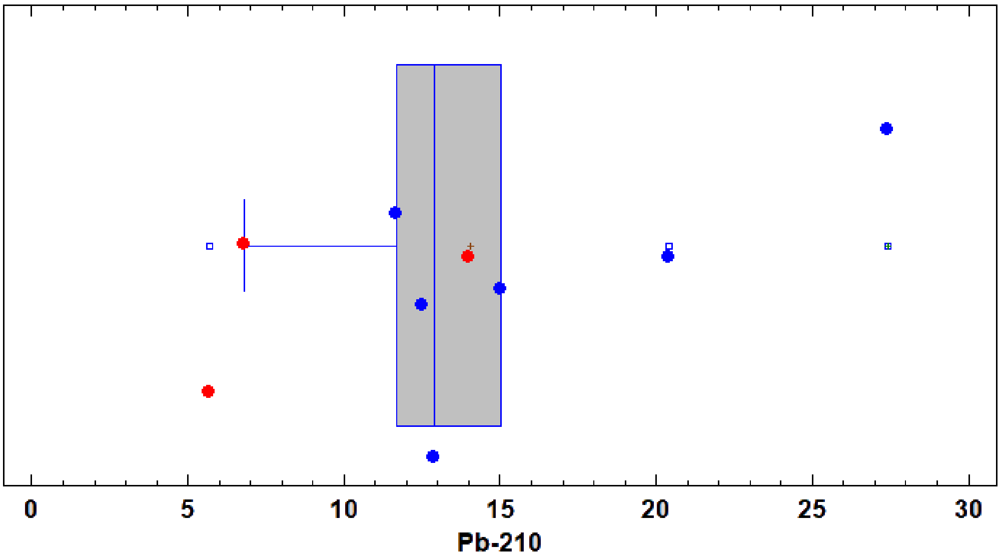


**Figure S2.8.** $^{210}$Pb activity concentrations box-and-whisker plot for ground mortar made with CEM II/A-S 42.5 N cement. The red dot shows the value for non-carbonated mortar and the blue dot shows the activity concentrations at different curing ages.

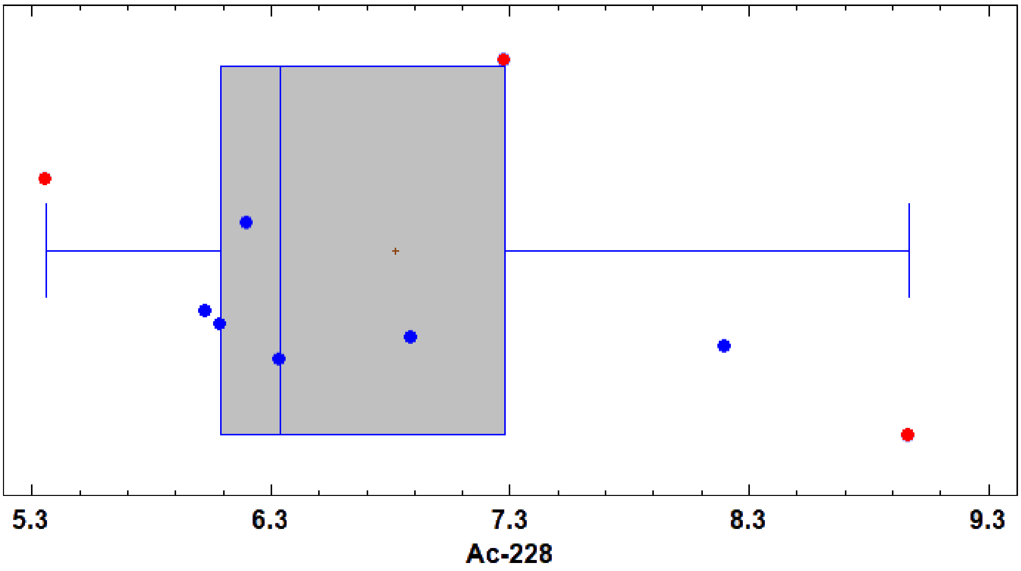


**Figure S2.9.** $^{228}$Ac activity concentrations box-and-whisker plot for ground mortar made with CEM II/A-S 42.5 N cement. The red dot shows the value for non-carbonated mortar and the blue dot shows the activity concentrations at different curing ages.

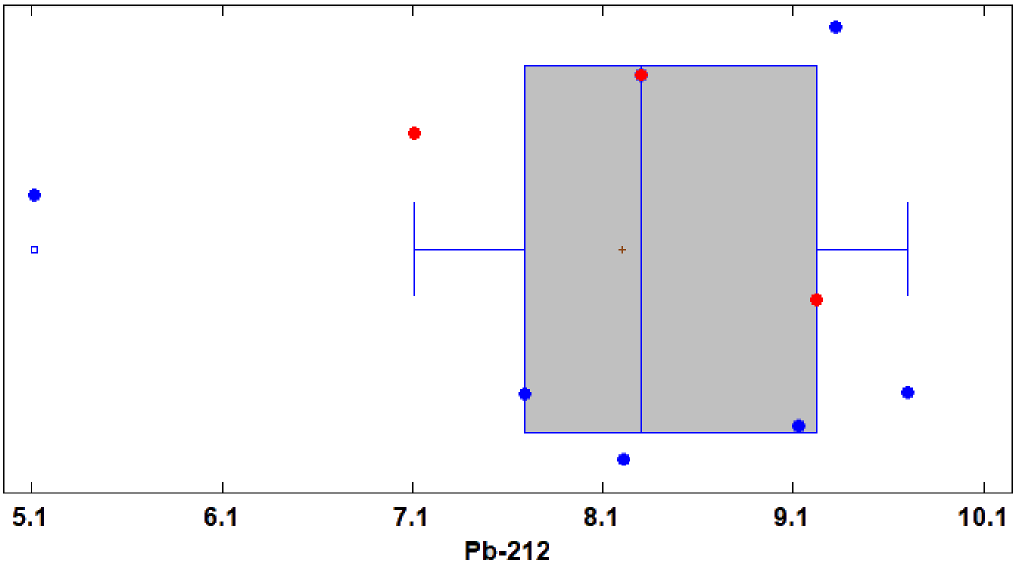


**Figure S2.10.** $^{212}$Pb activity concentrations box-and-whisker plot for ground mortar made with CEM II/A-S 42.5 N cement. The red dot shows the value for non-carbonated mortar and the blue dot shows the activity concentrations at different curing ages.

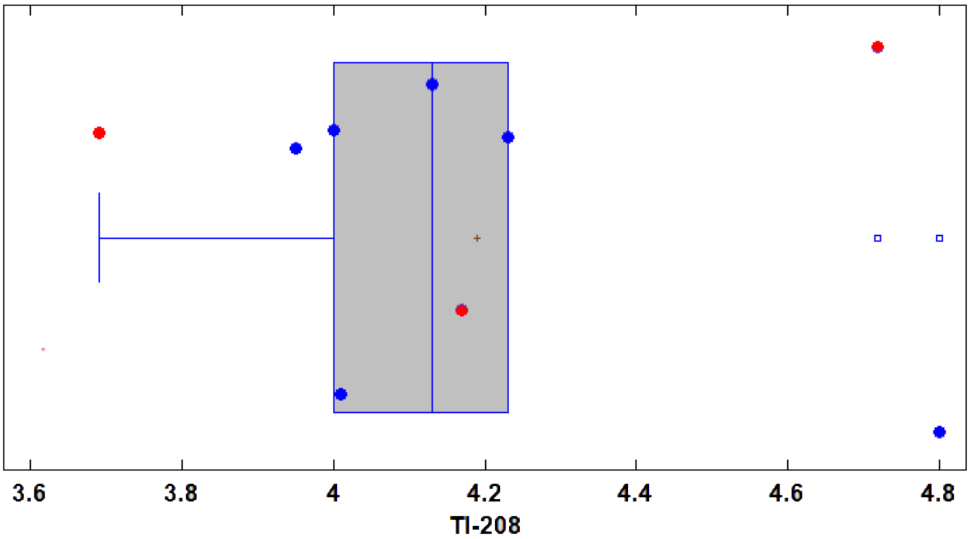


**Figure S2.11.** $^{208}$Tl activity concentrations box-and-whisker plot for ground mortar made with CEM II/A-S 42.5 N cement. The red dot shows the value for non-carbonated mortar and the blue dot shows the activity concentrations at different curing ages.

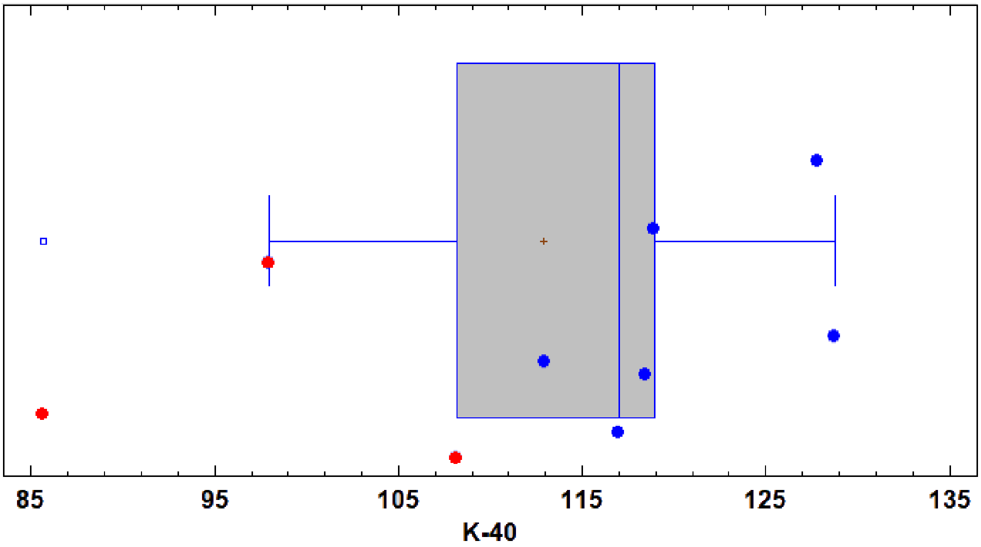


**Figure S2.12.** $^{40}$K activity concentrations box-and-whisker plot for ground mortar made with CEM II/A-S 42.5 N cement. The red dot shows the value for non-carbonated mortar and the blue dot shows the activity concentrations at different curing ages.

**Supplementary S3. Box-and-whisker and scatter plots for the radionuclides of mortars made with CEM III/A 42.5N cement.**

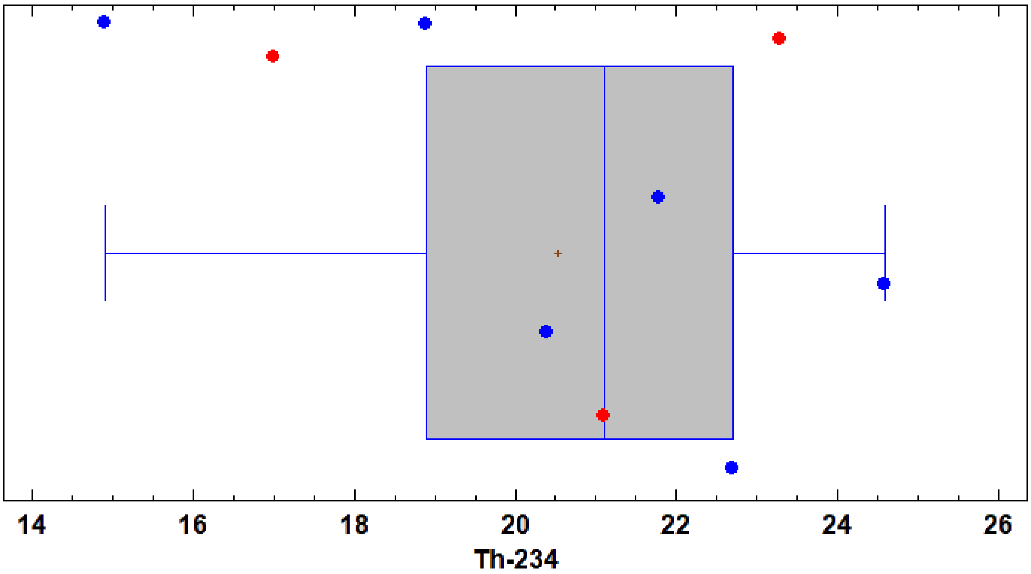


**Figure S3.1.** $^{234}Th$ activity concentrations box-and-whisker plot for ground mortar made with CEM III/A 42.5 N cement. The red dot shows the value for non-carbonated mortar and the blue dot shows the activity concentrations at different curing ages.

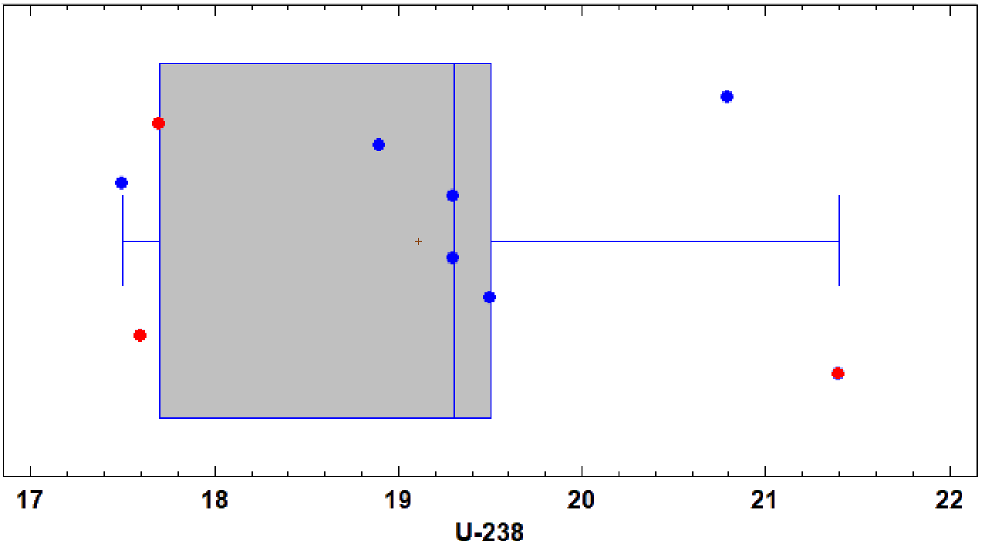


**Figure S3.2.** $^{238}U$ activity concentrations box-and-whisker plot for ground mortar made with CEM III/A 42.5 N cement. The red dot shows the value for non-carbonated mortar and the blue dot shows the activity concentrations at different curing ages.

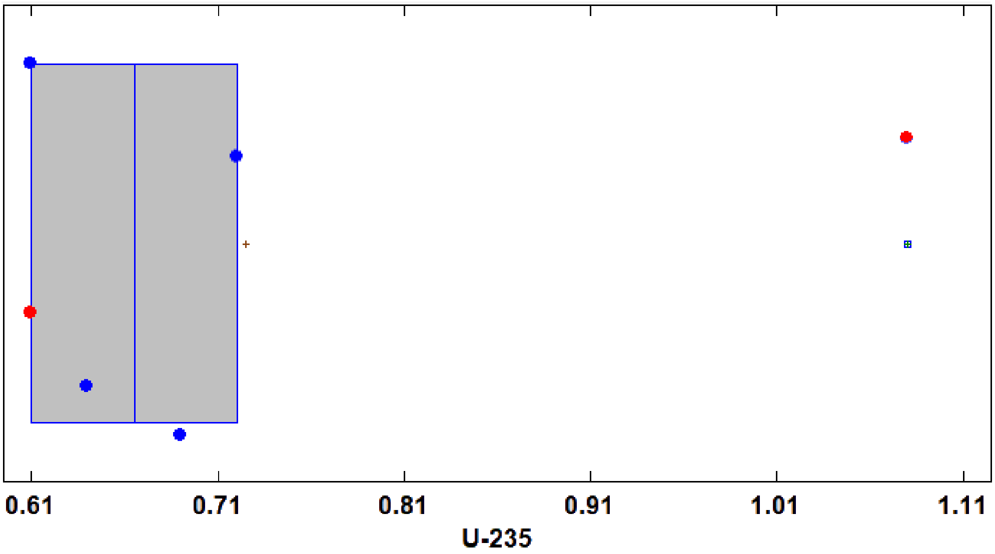


**Figure S3.3.** $^{235}U$ activity concentrations box-and-whisker plot for ground mortar made with CEM III/A 42.5 N cement. The red dot shows the value for non-carbonated mortar and the blue dot shows the activity concentrations at different curing ages.

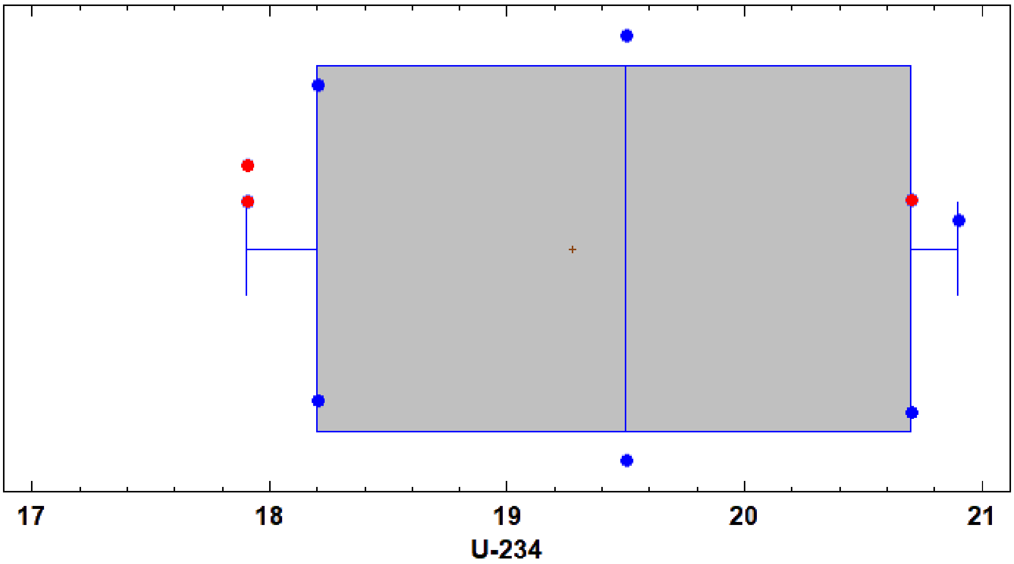


**Figure S3.4.** $^{234}U$ activity concentrations box-and-whisker plot for ground mortar made with CEM III/A 42.5 N cement. The red dot shows the value for non-carbonated mortar and the blue dot shows the activity concentrations at different curing ages.

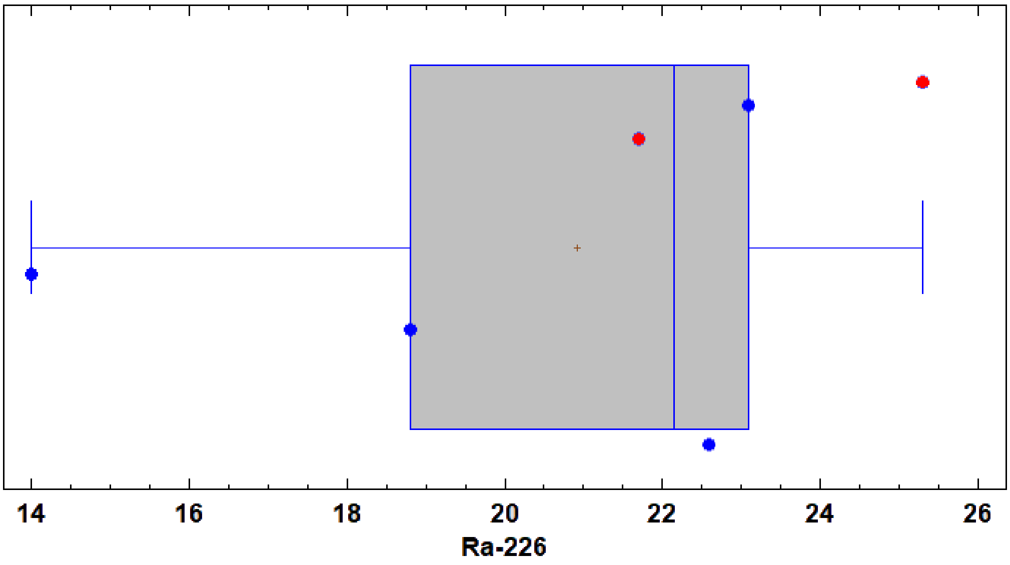


**Figure S3.5.** $^{226}$Ra activity concentrations box-and-whisker plot for ground mortar made with CEM III/A 42.5 N cement. The red dot shows the value for non-carbonated mortar and the blue dot shows the activity concentrations at different curing ages.

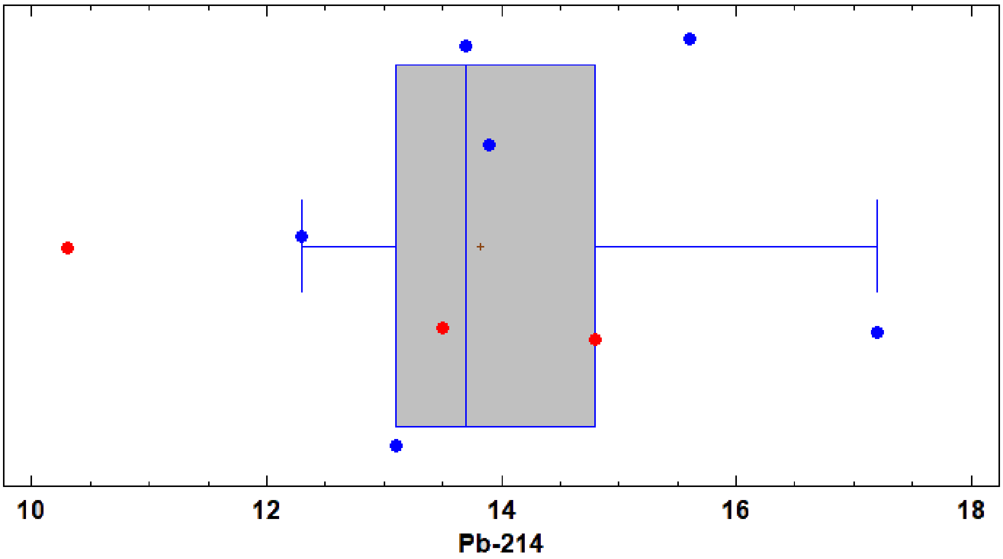


**Figure S3.6.** $^{214}$Pb activity concentrations box-and-whisker plot for ground mortar made with CEM III/A 42.5 N cement. The red dot shows the value for non-carbonated mortar and the blue dot shows the activity concentrations at different curing ages.

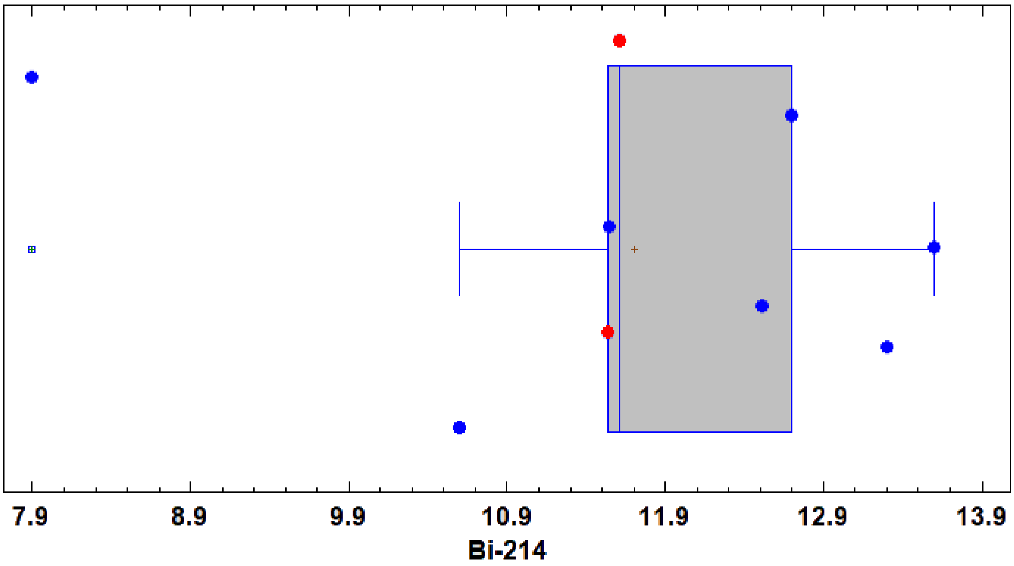


**Figure S3.7.** $^{214}$Bi activity concentrations box-and-whisker plot for ground mortar made with CEM III/A 42.5 N cement. The red dot shows the value for non-carbonated mortar and the blue dot shows the activity concentrations at different curing ages.

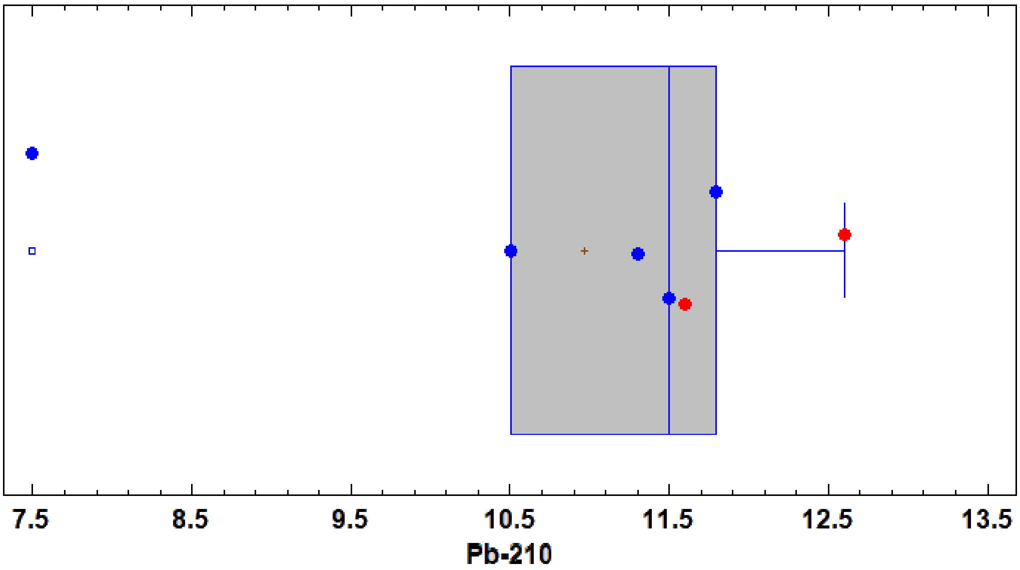


**Figure S3.8.** $^{210}$Pb activity concentrations box-and-whisker plot for ground mortar made with CEM III/A 42.5 N cement. The red dot shows the value for non-carbonated mortar and the blue dot shows the activity concentrations at different curing ages.

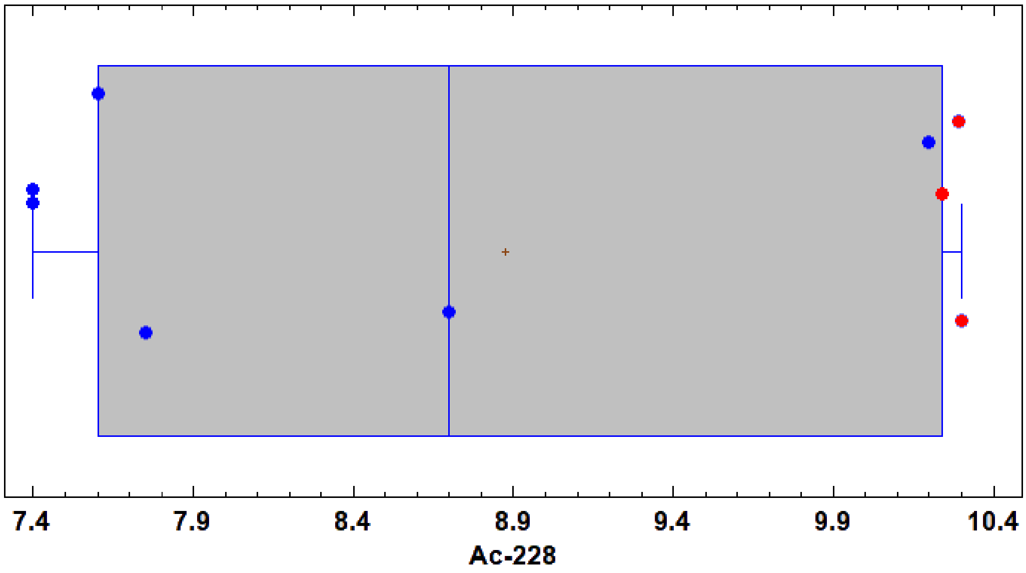


**Figure S3.9.** $^{228}$Ac activity concentrations box-and-whisker plot for ground mortar made with CEM III/A 42.5 N cement. The red dot shows the value for non-carbonated mortar and the blue dot shows the activity concentrations at different curing ages.

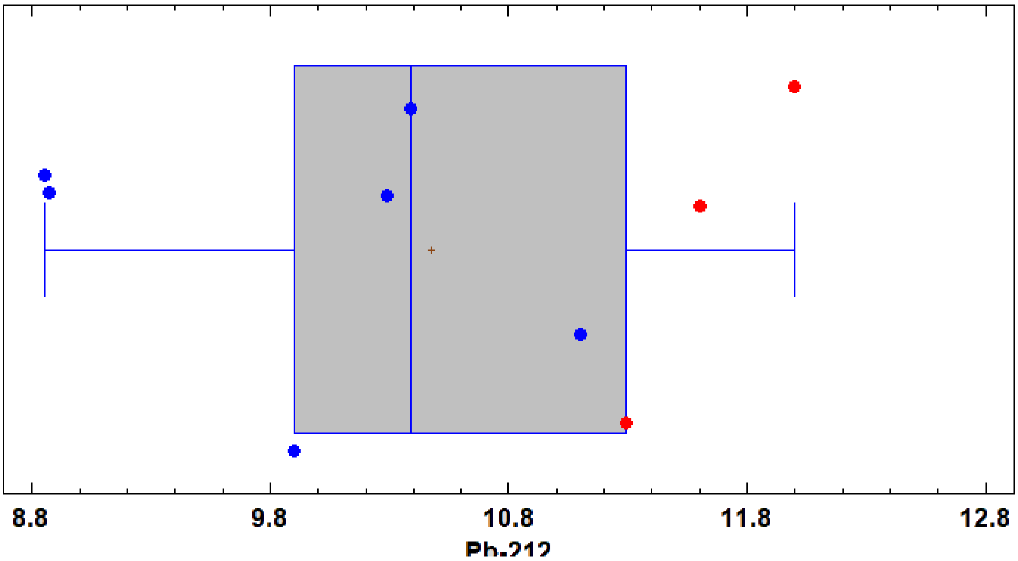


**Figure S3.10.** $^{212}$Pb activity concentrations box-and-whisker plot for ground mortar made with CEM III/A 42.5 N cement. The red dot shows the value for non-carbonated mortar and the blue dot shows the activity concentrations at different curing ages.

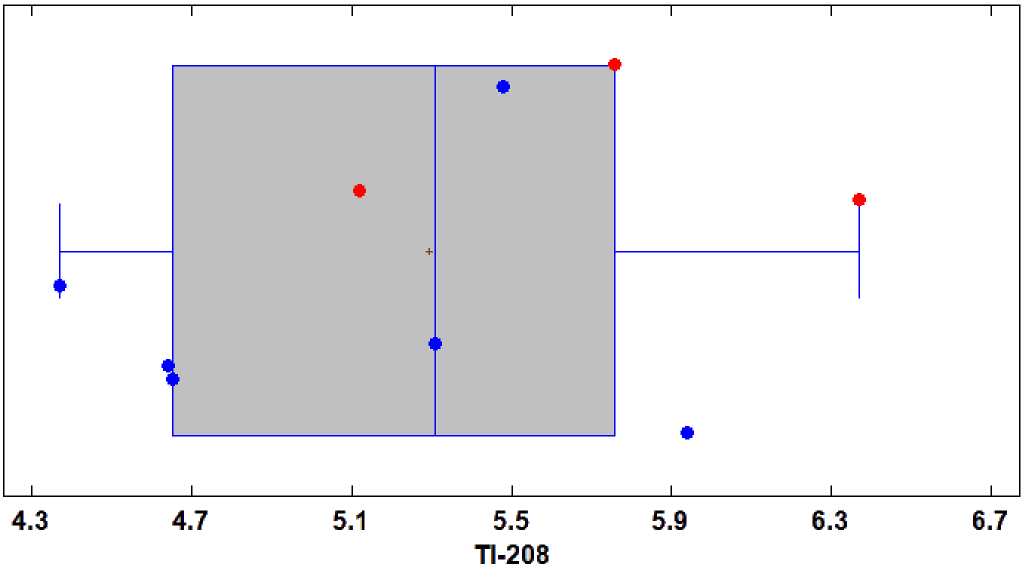


**Figure S3.11.** $^{208}$Tl activity concentrations box-and-whisker plot for ground mortar made with CEM III/A 42.5 N cement. The red dot shows the value for non-carbonated mortar and the blue dot shows the activity concentrations at different curing ages.

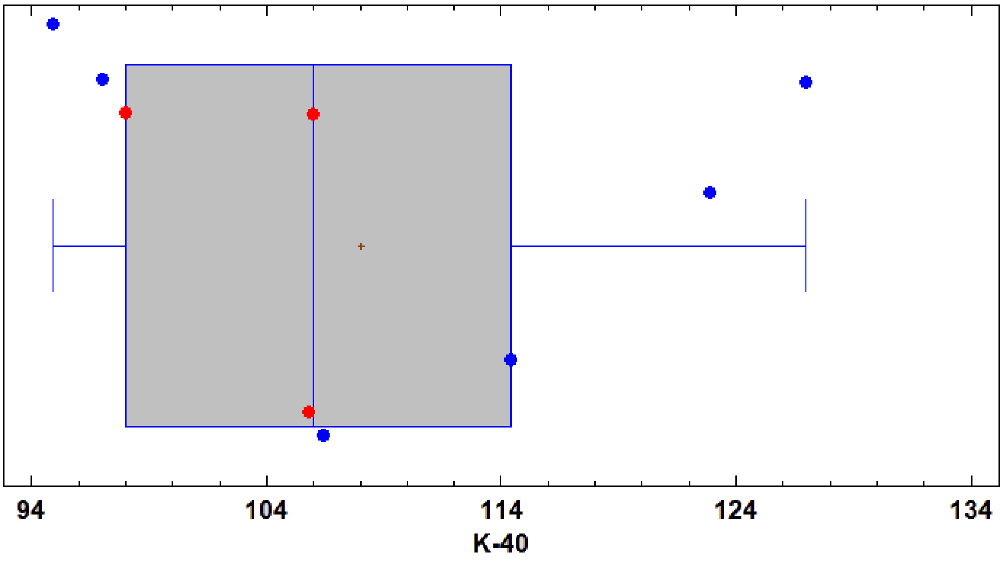


**Figure S3.12.** $^{40}$K activity concentrations box-and-whisker plot for ground mortar made with CEM III/A 42.5 N cement. The red dot shows the value for non-carbonated mortar and the blue dot shows the activity concentrations at different curing ages.

materials

**Supplementary S4. Comparison of the activity concentrations of the radioactive thorium series for the three tested mortars.**

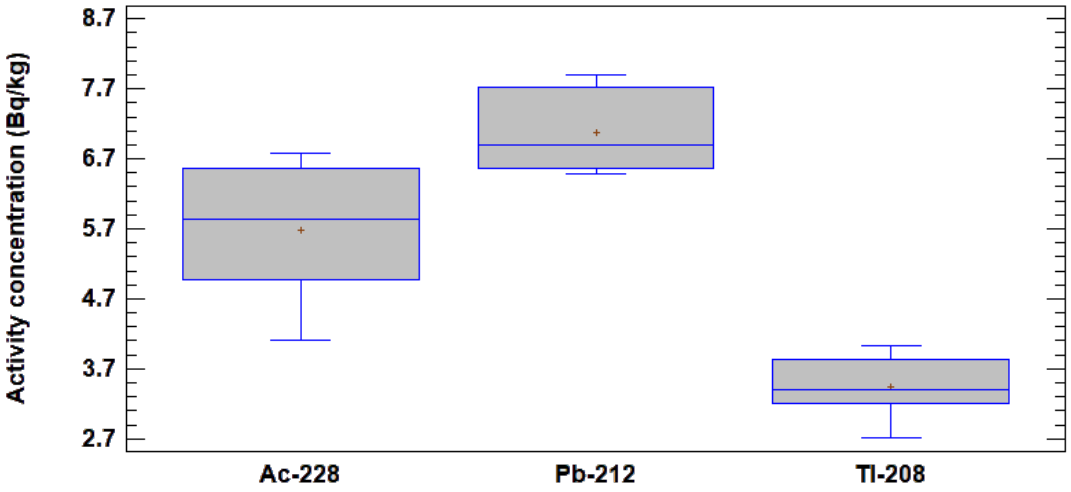


**Figure S4.1.** Activity concentration distributions of the radioactive thorium series for the mortar made with CEM I 52.5 R-SR 3 cement.

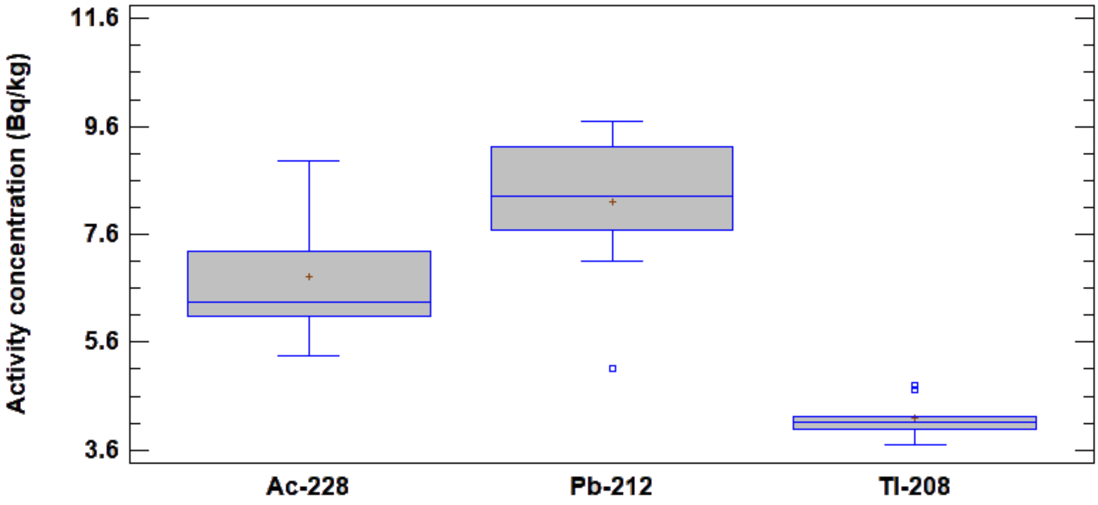


**Figure S4.2.** Activity concentration distributions of the radioactive thorium series for the mortar made with CEM II/A-S 42.5 N cement.

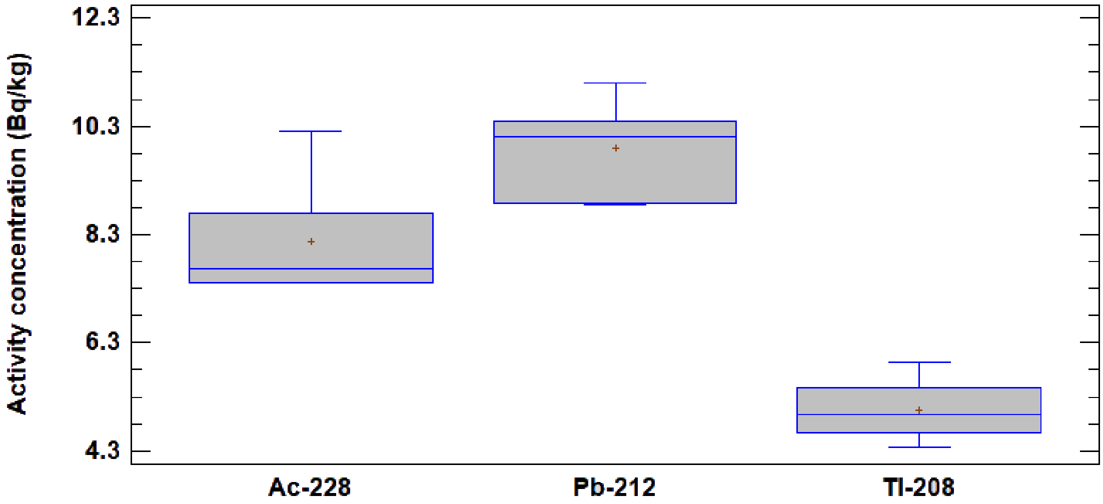


**Figure S4.3.** Activity concentration distributions of the radioactive thorium series for the mortar made with CEM III/A 42.5 N cement.
